# Acute Uncomplicated Febrile Illness in Children Aged 2-59 months in Zanzibar – Aetiologies, Antibiotic Treatment and Outcome

**DOI:** 10.1371/journal.pone.0146054

**Published:** 2016-01-28

**Authors:** Kristina Elfving, Deler Shakely, Maria Andersson, Kimberly Baltzell, Abdullah S. Ali, Marc Bachelard, Kerstin I. Falk, Annika Ljung, Mwinyi I. Msellem, Rahila S. Omar, Philippe Parola, Weiping Xu, Max Petzold, Birger Trollfors, Anders Björkman, Magnus Lindh, Andreas Mårtensson

**Affiliations:** 1 Department of Infectious Diseases, University of Gothenburg, Gothenburg, Sweden; 2 Department of Paediatrics, University of Gothenburg, Gothenburg, Sweden; 3 Malaria Research, Department of Microbiology, Tumour and Cell biology, Karolinska Institutet, Stockholm, Sweden; 4 Department of Medicine, Kungälv Hospital, Kungälv, Sweden; 5 Department of Family Health Care Nursing, University of California San Francisco, San Francisco, United States of America; 6 Zanzibar Malaria Elimination Programme, Ministry of Health, Zanzibar, Tanzania; 7 Department of Microbiology, Tumor and Cell biology, Karolinska Institutet, Stockholm, Sweden; 8 Aix Marseille University, UM63, WHO collaborative centre for rickettsioses and other arthropod borne bacterial diseases, Faculté de Médecine, Marseille, France; 9 Akademistatistik, Centre for Applied Biostatistics, Occupational and Environmental Medicine, University of Gothenburg, Gothenburg, Sweden; 10 Centre for Clinical Research Sörmland, Uppsala University, Uppsala, Sweden; 11 Department of Women’s and Children’s Health, International Maternal and Child Health Unit (IMCH), Uppsala University, Uppsala, Sweden; Naval Research Laboratory, UNITED STATES

## Abstract

**Background:**

Despite the fact that a large proportion of children with fever in Africa present at primary health care facilities, few studies have been designed to specifically study the causes of uncomplicated childhood febrile illness at this level of care, especially in areas like Zanzibar that has recently undergone a dramatic change from high to low malaria transmission.

**Methods:**

We prospectively studied the aetiology of febrile illness in 677 children aged 2–59 months with acute uncomplicated fever managed by IMCI (Integrated Management of Childhood Illness) guidelines in Zanzibar, using point-of-care tests, urine culture, blood-PCR, chest X-ray (CXR) of IMCI-pneumonia classified patients, and multiple quantitative (q)PCR investigations of nasopharyngeal (NPH) (all patients) and rectal (GE) swabs (diarrhoea patients). For comparison, we also performed NPH and GE qPCR analyses in 167 healthy community controls. Final fever diagnoses were retrospectively established based on all clinical and laboratory data. Clinical outcome was assessed during a 14-day follow-up. The utility of IMCI for identifying infections presumed to require antibiotics was evaluated.

**Findings:**

NPH-qPCR and GE-qPCR detected ≥1 pathogen in 657/672 (98%) and 153/164 (93%) of patients and 158/166 (95%) and 144/165 (87%) of controls, respectively. Overall, 57% (387/677) had IMCI-pneumonia, but only 12% (42/342) had CXR-confirmed pneumonia. Two patients were positive for *Plasmodium falciparum*. Respiratory syncytial virus (24.5%), influenza A/B (22.3%), rhinovirus (10.5%) and group-A streptococci (6.4%), CXR-confirmed pneumonia (6.2%), Shigella (4.3%) were the most common viral and bacterial fever diagnoses, respectively. Blood-PCR conducted in a sub-group of patients (n = 83) without defined fever diagnosis was negative for rickettsiae, chikungunya, dengue, Rift Valley fever and West Nile viruses. Antibiotics were prescribed to 500 (74%) patients, but only 152 (22%) had an infection retrospectively considered to require antibiotics. Clinical outcome was generally good. However, two children died. Only 68 (11%) patients remained febrile on day 3 and three of them had verified fever on day 14. An additional 29 (4.5%) children had fever relapse on day 14. Regression analysis determined C-reactive Protein (CRP) as the only independent variable significantly associated with CXR-confirmed pneumonia.

**Conclusions:**

This is the first study on uncomplicated febrile illness in African children that both applied a comprehensive laboratory panel and a healthy control group. A majority of patients had viral respiratory tract infection. Pathogens were frequently detected by qPCR also in asymptomatic children, demonstrating the importance of incorporating controls in fever aetiology studies. The precision of IMCI for identifying infections requiring antibiotics was low.

## Background

Despite substantial decline in global child mortality over the past two decades, over six million children die annually due to preventable or treatable illnesses approximately 50% of them in resource-limited settings in Africa [1].

The Integrated Management of Childhood Illness (IMCI) guidelines were developed to improve clinical management of children below five years of age by primary health care workers. It is comprised of simple algorithms based on symptoms and clinical signs that lead to a recommended treatment and/or management. However, the scientific base behind IMCI was generated over 20 years ago [2], and since then profound changes in the epidemiology of childhood febrile illness have occurred in many parts of Africa following e.g. the introduction of new vaccines, reduced malaria transmission, use of malaria rapid diagnostic tests (RDTs), and increasing antibiotic resistance [3].

Therefore, it is necessary to re-evaluate the aetiology of acute uncomplicated childhood fever and also to assess whether IMCI remains a reliable tool for identification of children with infections presumed to benefit from antibiotic treatment [4].

The complexity of fever aetiology has been highlighted by the application of molecular assays for detection of multiple pathogens [5,6]. The frequent detection of nucleic acids from pathogens among both patients and asymptomatic children [7,8] points at the need to include healthy controls in fever aetiology studies. However, comprehensive studies of this type, especially those from Africa are lacking.

We therefore studied the aetiology and outcome of acute uncomplicated febrile illness in children 2–59 months seeking care at primary health care level in Zanzibar, a malaria pre-elimination setting of sub-Saharan Africa, using both point-of-care tests, urine cultures, confirmatory chest X-ray (CXR) investigation (in patients with IMCI-pneumonia classification), as well as multiplex quantitative (q)PCR investigations of nasopharyngeal (NPH) and rectal swabs from both patients and healthy controls. We also assessed the utility of IMCI in identification of infections presumed to require antibiotics.

## Methods

### Study design and study site

This was a prospective descriptive health facility based study conducted in North A District, Zanzibar, Tanzania between April-July 2011 that followed children with acute uncomplicated febrile illness for 14 days. A healthy community control group was recruited for comparison during the same study time period.

Zanzibar has a tropical climate with seasonal rainfalls biannually. Malaria positivity rate has declined dramatically over the past decade from approximately 40% to 1–2% in febrile patients after wide-scale deployment of malaria control interventions [9]. The study district is mainly rural with approximately 100,000 inhabitants. Public health care is delivered through 12 primary health care units and one primary health care centre (Kivunge). In addition to first-level outpatient care, Kivunge primary health care centre has facilities for basic inpatient care and laboratory services. It was selected as study site based on its central location in the district, 24-hour service, presence of a research laboratory, and radiology equipment.

### Study staff

The study staff included clinicians (clinical officers with prescription rights), qualified nurses and laboratory technicians. The clinicians had been trained in IMCI management, in particular regarding integration of malaria RDT as previously described [10] and measurement of respiratory rate.

### Patient enrolment

Children presenting at the study site were screened for eligibility. Up to 15 patients were enrolled daily Monday-Saturday. Inclusion criteria were: age 2–59 months; acute uncomplicated fever defined as history of fever in the preceding 24 hours (information from caretaker) and/or verified fever (axillary temperature of ≥37.5°C by electronic thermometer); and written informed proxy consent from an accompanying caretaker. Exclusion criteria were: signs of severe disease (according to IMCI); previous study enrolment in the last 28 days; and reported inability to return for follow-up. Data were documented in a structured IMCI-based questionnaire.

### Patient investigations and clinical management

**Fig 1:**
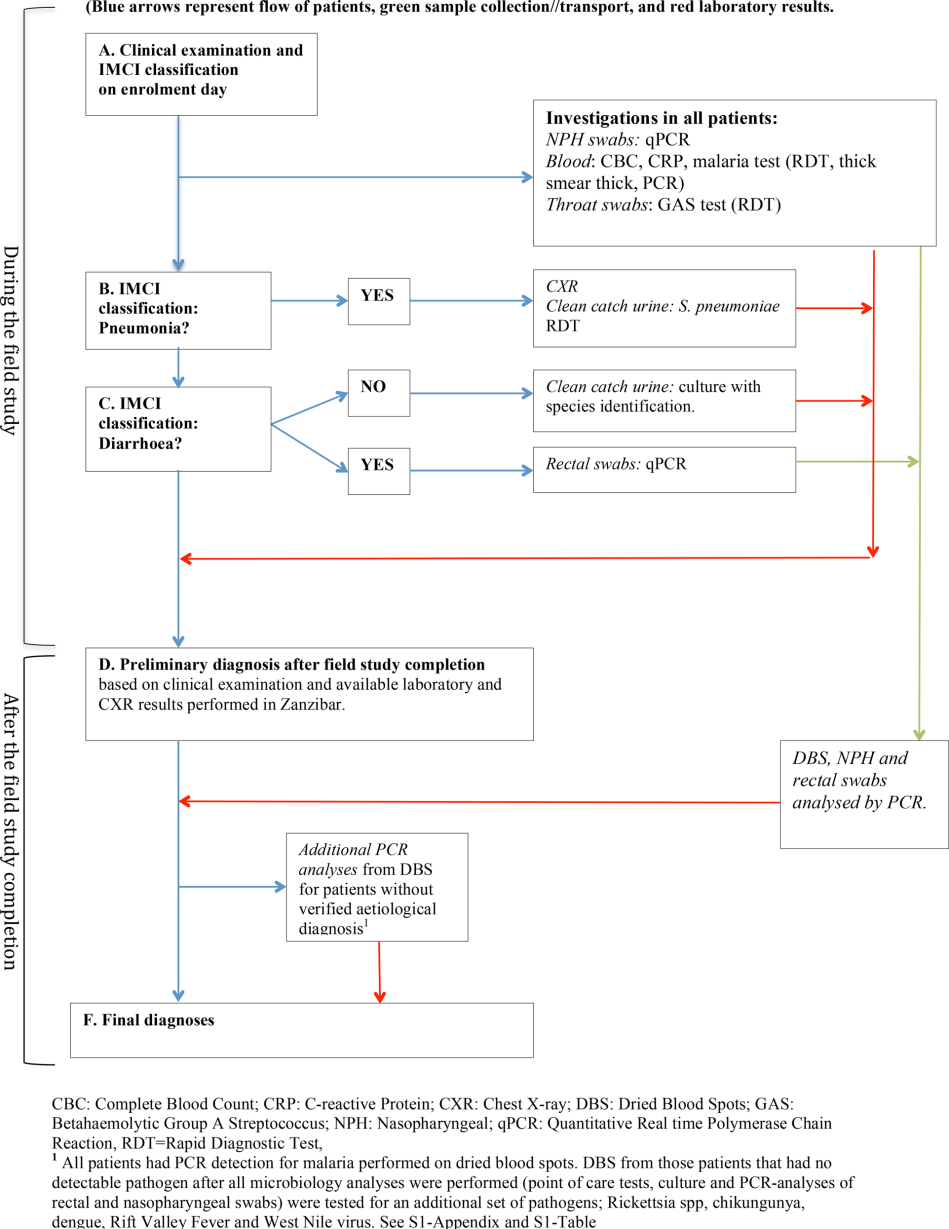
Flow chart from investigations and management to final diagnoses.

**Fig 2:**
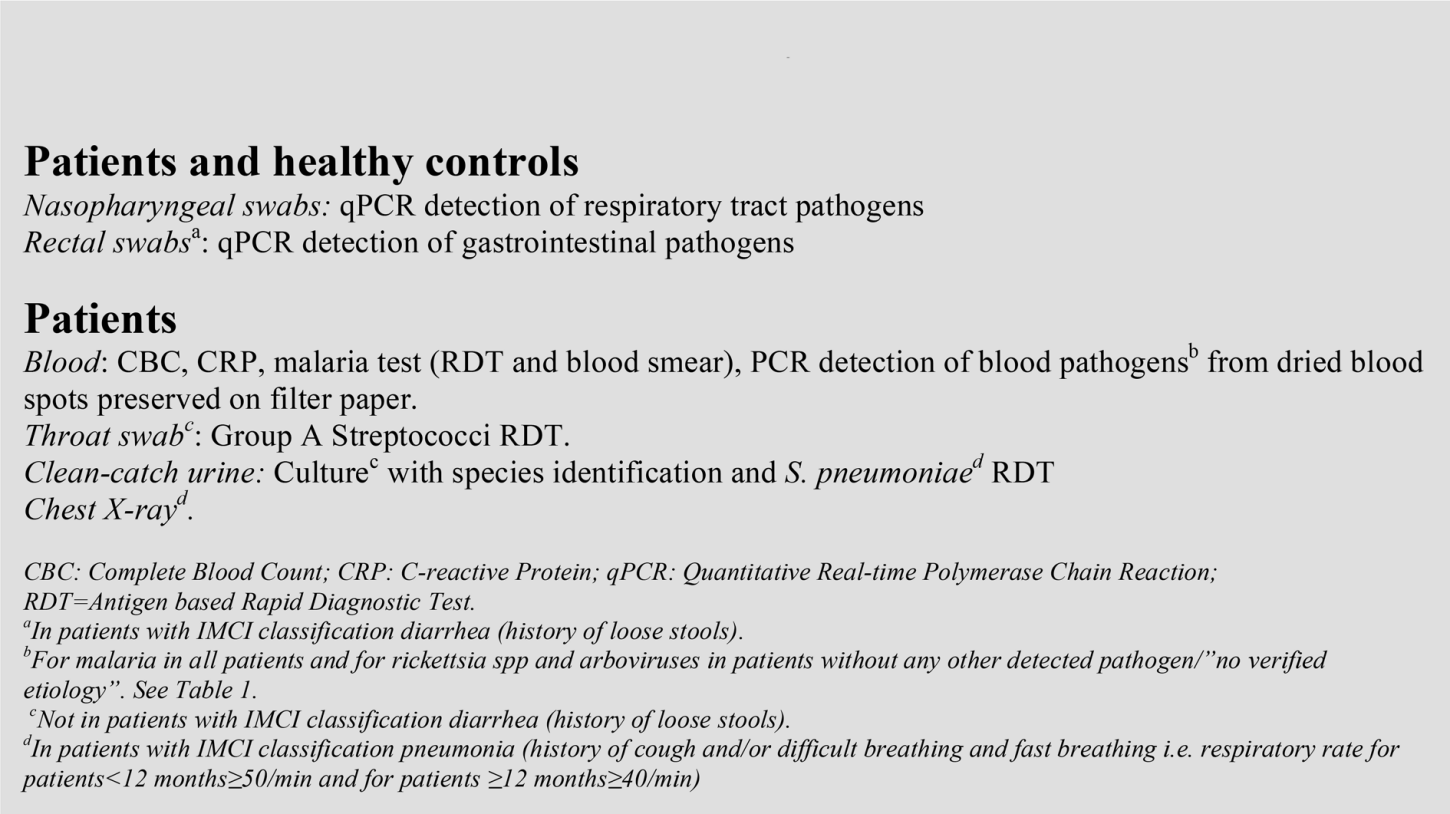
Baseline specimen collection and investigations.

After enrolment, (defined as day 0) a study nurse performed a malaria RDT and a study clinician conducted IMCI assessment and prescribed treatment. This was followed by pre-defined investigations including point-of-care tests, sampling for microbiology analyses, and CXR, some performed in all patients others in patients with certain clinical features identified through IMCI management (Figs 1 and 2). Participants with pre-defined abnormal laboratory results and/or signs of severe disease were discontinued and referred for further clinical management (Table 1).

**Table 1:** Study definitions.

Definition	Explanation
***Classification***	
Fast breathing	>50 breaths/min for patients aged 2–11 months; >40 breaths/min for patients aged 12–59 months
Fever	Verified fever or fever by history in the preceding 24 hours
Verified fever	≥37.5°C, axillary temperature
Severe disease	Symptoms/signs according to the IMCI guidelines
Abnormal laboratory values	CRP >200 mg/L; WBC >35*10^9/L; PLC <20*10^9/L; Hb<6 g/dL^a^
Infections Requiring Antibiotics (InfRA)	Infection expected to benefit from systemic antibiotics, defined by the study team in retrospect
IMCI indicating antibiotic treatment (IMCIAB)	Classification in IMCI that indicates systemic antibiotics
IMCI pneumonia	Cough and/or difficult breathing and fast breathing
Serious bacterial infection	CXR-confirmed pneumonia and/or urinary tract infection
***Study outcome***	
Discontinued due to withdrawal of consent	Withdrawal of consent by the care taker for participation in the study
Discontinued due to severe disease or severe laboratory values	Patient developed severe disease or severe laboratory values within the 14-day follow-up period
Death	The patient died within the 14-day follow-up period
Incomplete follow up data	No data on first or second scheduled follow-up.
Lost to follow up	Patient did not return for the final 14 day scheduled follow-up visit and could not be traced
Completed	Patient completed all follow-ups and did not fulfil any of the above-mentioned outcomes.
***Resolution of fever during follow-up***	
Follow-up 1	Patient follow-up on day 4 (+/-2 days). Primarily based on IMCI
Follow-up 2	Patient follow-up on day 14 (+/-2 days). Study outcome follow-up
Early resolution of fever	No verified fever on follow-up 1 and 2
Late resolution of fever	Verified fever on follow-up 1. No verified fever on follow-up 2
Relapse	No fever on follow-up 1. Verified fever on follow-up 2
No resolution of fever	Verified fever on follow-up 1 and 2

^a^ Patients not meeting these criteria but with a CRP of >150 mg/L, WBC >25*10^9/L, or PLC<50*10^9/L were followed up the next day.

ARI, Acute respiratory tract infection

CRP, C-reactive protein

CXR, Chest X-ray

GE, gastroenteritis

Hb, haemoglobin

IMCI, Integrated Management of Childhood Illnesses Guidelines

InfRA, Infections requiring systemic antibiotic treatment

PLC, platelet counts

RDT, Rapid Diagnostic Test

WBC, White blood cell counts.

All study investigations are presented in Fig 1, Fig 2 and S1A–S1L Appendix. Sampling, storage and performance of all diagnostics were done according to manufacturers’ instructions and study specific standard operating procedures. Some investigations were conducted on-site or in Mnazi Mmoja referral hospital. The remaining laboratory analyses were performed in Sweden and France after study completion. CXRs were performed and interpreted according to World Health Organization (WHO) standards with quality control readings done in Sweden [11].

### Patient follow-up

**Fig 3:**
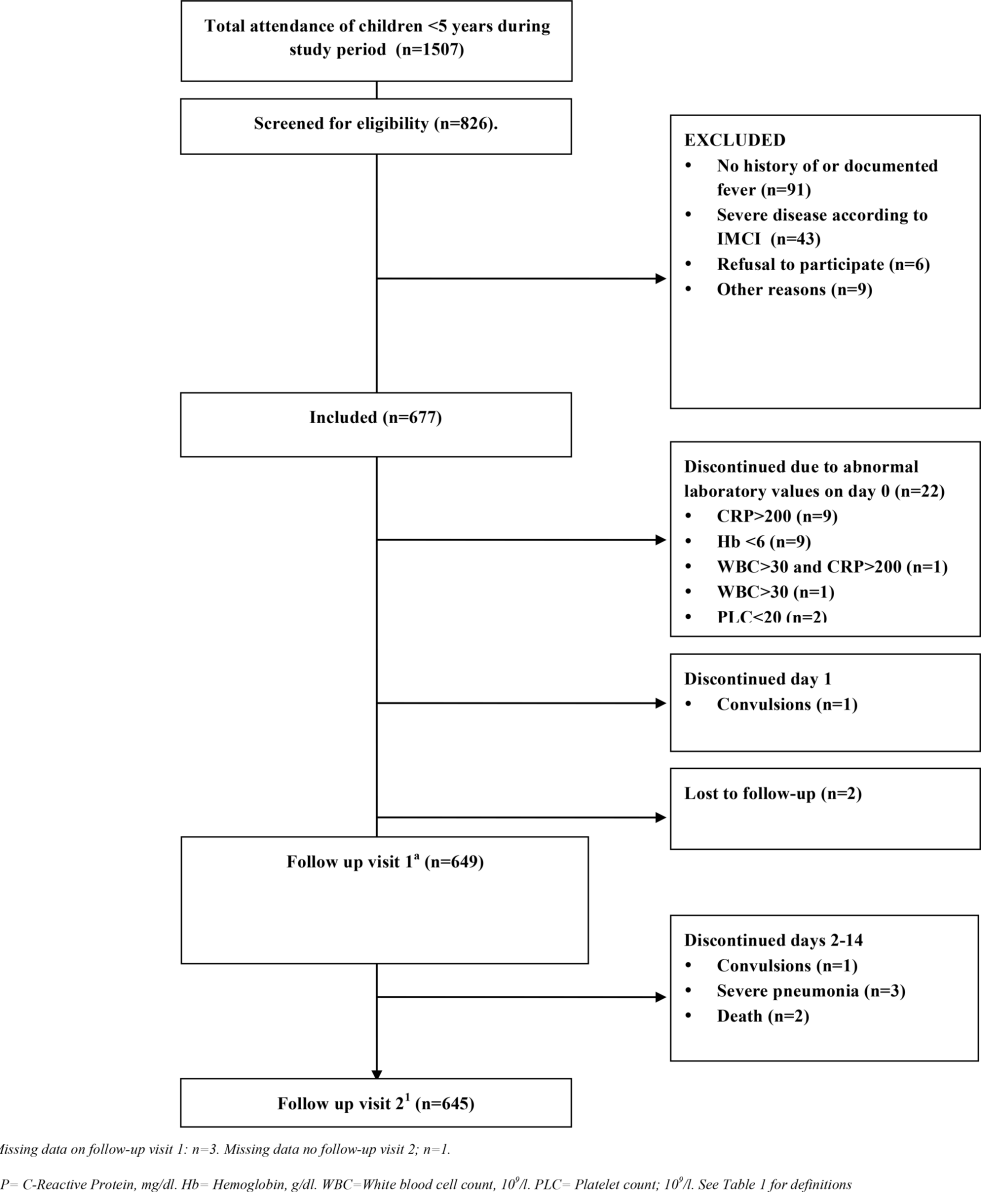
Flow of patients through the study.

Patients had a first scheduled follow-up on day four (+/-2 days) and a second on day 14 (+/-2 days) for evaluation of fever outcome (Fig 3). Caretakers were instructed to return with their children in case any signs of severe disease occurred. On every follow-up visit a study clinician performed clinical reassessment including axillary temperature. If a child did not return for a scheduled follow-up, it was actively traced at home. Any patient with symptoms/signs of severe disease, predefined abnormal laboratory values or withdrawal of consent during the 14-day follow-up was discontinued from the study (Fig 3).

### Healthy controls

Healthy controls (hereafter referred to as “controls”), defined as children aged 2–59 months with no history of diarrhoea, cough, running nose or fever (by history and/or electronic axillary temperature <37.5°C) in the preceding ten days were recruited during the same study time period. Recruitment aimed at a representative distribution of age, sex and geography. Based on previous health facility data, eight villages in the catchment area with high attendance to the study primary health care centre were identified. Each study week, one of these eight villages was visited and asymptomatic children were identified through house-to-house screening. Eligible children, maximum two per household, provided NPH and rectal swabs for qPCR-analyses (Fig 2).

### Assessment of final diagnoses

Criteria for the respective final diagnoses were defined by two study investigators (paediatricians) based on all available clinical and radiology/laboratory data from day 0 (Tables 1 and 2, Fig 1) including the multiple regression analysis of qPCR results described further in S1J Appendix. Subsequently these criteria were applied on each patient. The diagnoses were categorized into three groups: 1) more probable causes of fever, 2) less probable causes of fever, and 3) “no verified aetiology”. If a more probable diagnosis was identified, all less probable diagnoses were ignored for that patient, but within one group each patient could receive more than one diagnosis.

**Table 2:** Diagnostic criteria and study interventions.

Final diagnosis	Criteria in clinical history or examination	Investigation criteria	InfRA	Intervention
**More probable cause of fever**				
Acute ear infection	Reported ear discharge≤14 days	no	Yes	No
Chronic ear infection	Reported ear discharge>14 days	no	Yes	No
Malaria	no	Malaria RDT or microscopy or PCR +	NO	Yes^a^
Measles	no	Morbilli PCR +	No	No
Whooping cough	Common cold	Pertussis PCR: Ct<35	Yes	No
CXR-confirmed pneumonia	Fast breathing	Chest X-ray shows end-point consolidation^b^	Yes	Yes^c^
Streptococcal skin infection (scarlet fever or impetigo)	Scarlatine skin rash or impetigo	RDT GAS+	Yes	Yes^d^
Streptococcal tonsillitis	Sore throat/tonsillitis/lymphadenitis	RDT GAS+	Yes	Yes^d^
Urinary tract infection	No diarrhoea	Positive urine culture^e^ AND any of the following urine-leukocytes (2+) OR urine-nitrite +	Yes	Yes^f^
Pyelonephritis	No diarrhoea	Same criteria as Urinary tract infection AND CRP≥50.	Yes	Yes^f^
Dysentery^g^	History of bloody stools	PCR+^h^^i^	If *Shigella* PCR+	No
GE diagnoses^g^	≥3 loose stools per day	PCR+^h^	If *Shigella* PCR+	No
ARI diagnoses^j^	no	PCR+^k^	If *Ch*. *pneumoniae* PCR+	No
**Less probable cause of fever**				
Possible streptococcal infection	no	RDT GAS+	Yes	Yes^d^
GE diagnoses^g^	≥3 loose stools per day	PCR+ ^i^	If *Shigella* PCR+	No
ARI diagnoses^j^	no	PCR+^m^	No	No
No defined aetiology	Not fulfilling any group 1 or group 2 diagnoses	no	No	Yes

^a^ In cases of positive malaria microscopy, undetected by RDT, these patients were to be treated with antimalarials.

^b^ WHO defined radiology criteria for diagnosis of pneumonia (Cherian et al, 2005)

^c^ If suspected severe disease on CXR, patients were referred to a paediatric specialist

^d^ All RDT GAS positive were treated with Penicillin V if not already treated with an equivalent antibiotic

^e^ Significant growth of urinary pathogens on urine culture, ≥10^4 cfu/ml.

^f^ Patients with positive urine cultures were treated with an antibiotic corresponding to the susceptibility pattern

^g^ Rectal swab PCR.

^h^ More likely cause of disease pathogens: Norovirus GII, rotavirus, *Cryptosporidium* with Ct<35, enterotoxigenic *E*. *coli* (heat stable toxin) with Ct<31, and *Shigella* spp with Ct<30.

^i^ Less likely cause of disease pathogens Adenovirus, *Campylobacter* and sapovirus, *Cryptosporidium* with Ct≥35, enterotoxigenic E. Coli (heat lable toxin), enterotoxigenic *E*. *coli* (heat stable toxin) with Ct≥31, *Shigella* with Ct≥30

^j^ Nasopharyngeal swab PCR

^k^ More probable cause of disease pathogens: Enterovirus, influenza A virus, influenza B virus, RSV

^m^ Less probable cause of disease pathogens: Adenovirus, bocavirus, *Chlamydophila pneumoniae*, coronavirus, metapneumovirus, parainfluenza virus, parechovirus and rhinovirus.

For all nasopharyngeal and rectal swab PCRs: Ct-values<40 were disregarded in the final analysis.

### Assessment of infections requiring antibiotics

Study definitions are outlined in Table 1. Infections requiring antibiotics were defined retrospectively as those final diagnoses presumed to benefit from antibiotics based on WHO recommendations [12]. The association between these infections and both actually prescribed antibiotics and IMCI indication for antibiotics was assessed.

### Sample size calculation, study endpoints, data management and statistical analysis

This was an exploratory study, which precluded a sample size calculation. However, we estimated that a sample of 650 patients would be sufficient to obtain a representative classification of fever causes according to IMCI, and that at least 150 controls would be required to make comparisons of microbiological findings obtained by PCR analysis of nasopharyngeal and rectal specimens. Data were double entered in CSPro, validated and exported to STATA® 12 where all statistical analyses were performed. Frequencies, proportions and odds ratios (ORs) were calculated with 95% confidence intervals (CI). P-values <0.05 were considered statistically significant. Fisher’s exact test and exact binomial test was used for binary data and proportions, two-sample t-test for comparisons of means, and Mann-Whitney-U test for median comparisons. In a univariate and multivariate logistic regression we assessed the association between CXR-confirmed pneumonia and the continuous and binary variables outlined in Table 3. WBC (>20x 10^9/L) and CRP cut-off values (<20 and >80 mg/L), were chosen to concur with published literature [13].

**Table 3:** Factors associated with CXR-confirmed pneumonia among patients with IMCI pneumonia.

	CXR-confirmed pneumonia	No CXR-confirmed pneumonia	Unadjusted OR (CI)	*p*	Adjusted OR (CI)	*p*
	(n = 42)	(n = 300)				
**Continuous variables**						
Mean age, months (SD) / OR (CI)	20.2 (1.97)	16.4 (0.63)	1.03 (1.00–1.05)	0.041	1.03 (1.0–1.1)	0.051
Mean CRP, mg/L (CI) / OR (CI)	61.5 (9.9)	26.0 (1.7)	1.02 (1.01–1.02)	<0.0001	1.02 (1.01–1.03)	<0.0001
Mean WBC, 10^9^/L / OR (CI)	14.5 (1.1)	12.3 (0.2)	1.08 (1.0–1.2)	0.009	1.02 (0.9–1.1)	0.6
**Discrete variables**						
Child perceived as having fast breathing by care taker	85% (73–96%)	75% (70–80%)	1.8 (0.7–4.5)	0.2	1.9 (0.7–5.2)	0.19
Temperature ≥39.0°C (CI)	11% (7–15%)	14% (4–25%)	1.3 (0.5–3.5)	0.53	1.2 (0.4–4.0)	0.31
CRP^a^ <20 mg/L (CI)	36% (21–50%)	62% (56–67%)	0.3 (0.2–0.7)	0.002	^b^	^b^
CRP^a^ >80 mg/L (CI)	24% (11–37%)	5% (3–7%)	5.9 (2.4–14.3)	<0.0001	^b^	^b^
WBC >20 x 10^9^/L (CI)	10% (6–18%)	6% (3–9%)	1.6 (0.5–5.1)	0.4	^b^	^b^
Pneumococcal urine antigen positivity (CI)	59% (43–74%)	58% (53–64%)	1.0 (0.5–1.9)	0.99	1.3 (0.6–2.9)	0.48
Pneumococcal NPH PCR positivity (CI)	88% (78–98%)	86% (82–90%)	1.2 (0.4–3.2)	0.75	^b^	^b^
RSV NPH PCR positivity (CI)	26% (13–40%)	35% (30–40%)	0.7 (0.3–1.4)	0.26	0.7 (0.3–1.6)	0.36
IfA or IfB NPH PCR positivity (CI)	14% (4–25%)	18% (14–23%)	0.7 (0.3–1.8)	0.52	0.6 (0.2–1.8)	0.38

28 of 387 patients with IMCI pneumonia had no CXR performed. Another 17 patients were excluded from analysis because CXR was of too poor quality (n = 16) or missing (n = 1) CXR.

^a^CXR-confirmed pneumonia was seen in 8% (15/200) of patients with CRP <20 mg/L (p = 0.002), and in 40% (10/25) of those with CRP >80 mg/L (p<0.0001).

^b^Not included in final multivariate regression analysis

### Ethical considerations

The study was conducted in accordance with the Declaration of Helsinki and approved by the Zanzibar Medical Research Ethics Committee (ID: ZAMREC/0001/April/010) and Regional Research Ethics Committees in Stockholm (ID:2009/387-31/2), and Gothenburg (ID:266–10), Sweden. A written informed proxy consent from an accompanying caretaker was obtained and subsequently recorded on a consent form for all patients as well as healthy controls recruited in the study. An assistant medical officer ensured that laboratory results requiring medical intervention (Table 2) and/or referral were managed accordingly. Clinical management, treatments and referrals were provided free of charge to all study participants. No participant received any financial incentive, except for travel expenses for patients. Study registration on clinicaltrials.org (NCT01094431) was done before data acquisition.

## Results

### Participant characteristics, IMCI classifications and fever outcome

**Fig 4:**
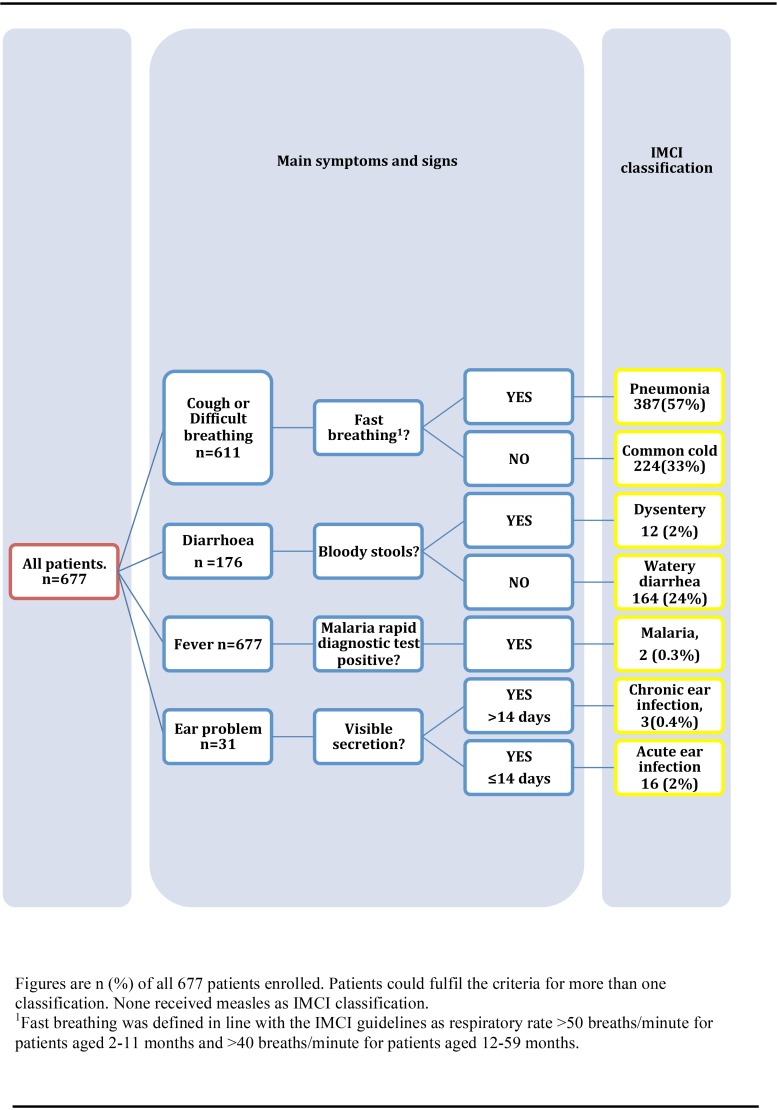
IMCI classifications associated with fever.

Baseline characteristics of the 677 patients and 167 controls are presented in Table 4. Forty-two percent (286/677) of patients had verified fever at enrolment. All patients were classified according to IMCI (Fig 4). More than one IMCI classification was assigned to 166 (25%) patients. Of 642 (96%) patients who completed both of the follow-up visits (Fig 3), 68 (11%) remained febrile on the first follow-up visit, and 3 of them had verified fever on day 14. An additional 29 (4.5%) children had verified fever relapse on the second follow-up visit.

**Table 4:** Demographic, socioeconomic and clinical characteristics of study participants.

Characteristics	Patients	Healthy controls
Number of enrolled participants	677 (100%)	167 (100%)
Male	352 (52%)	86 (51%)
Female	325 (48%)	81 (49%)
**Age**		
Median age months (IQR)	14 (9–24)	24 (12–36)
2–11 months	232 (34%)	32 (19%)
12–23 months	231 (34%)	42 (25%)
24–35 months	109 (16%)	41 (25%)
36–59 months	105 (16%)	52 (31%)
Median reported fever duration; days (IQR); (range)	3 (2–4)^a^; (1–14)	
**Care taker level of education**		
No school education	253 (37%)	
≤6 years education	92 (14%)	
>6 years education	317 (47%)	
Breastfeeding children <24 months	434 (94%)^a^ ^b^	
Fully immunized^c^ >11 months	420 (94%)^d^	
Antibiotics consumed before study inclusion	56 (8%)	
Paracetamol consumed before study inclusion	375 (55%)	
Underweight; % below -2SD (CI)	28.3 (24.9–31.8)	
**Temperature**		
Median temperature (IQR)	37.3 (36.8–38.0)	
36.0–37.4	375 (55%)	
37.5–39.0	240 (35%)	
>39.0	46 (7%)	
**Most common main complaints**		
Fever	644 (95%)	
Cough	580 (86%)	
Runny nose	455 (67%)	
Diarrhoea	158 (23%)	
Abdominal pain	35 (5%)	
Vomiting	34 (5%)	
Ear pain	21 (3%)	
Loss of appetite	17 (3%)	
**Antibiotic prescription on day 0**	500 (74%)	
One type of antibiotic	394 (79%)	
Two types of antibiotics	103 (21%)	
Three types of antibiotics.	3 (0.1%)	
Beta lactam antibiotics / parenteral benzyl penicillin	470 (93%) / 112 (17%)	
Trimethoprim/sulfamethoxazole	74 (11%)	
Other types of antibiotics	95 (14%)	

Denominators vary due to missing data (n≤10 if not indicated)

IQR = Interquartile range

^a^Missing data n>10, ≤50

^b^Denominator patients<24 months: n = 463

^c^ BCG, OPV3, Pentavalent/DPT3, measles

^d^Denominator patients>11 months: n = 445.

### Detection of pathogens

**Fig 5:**
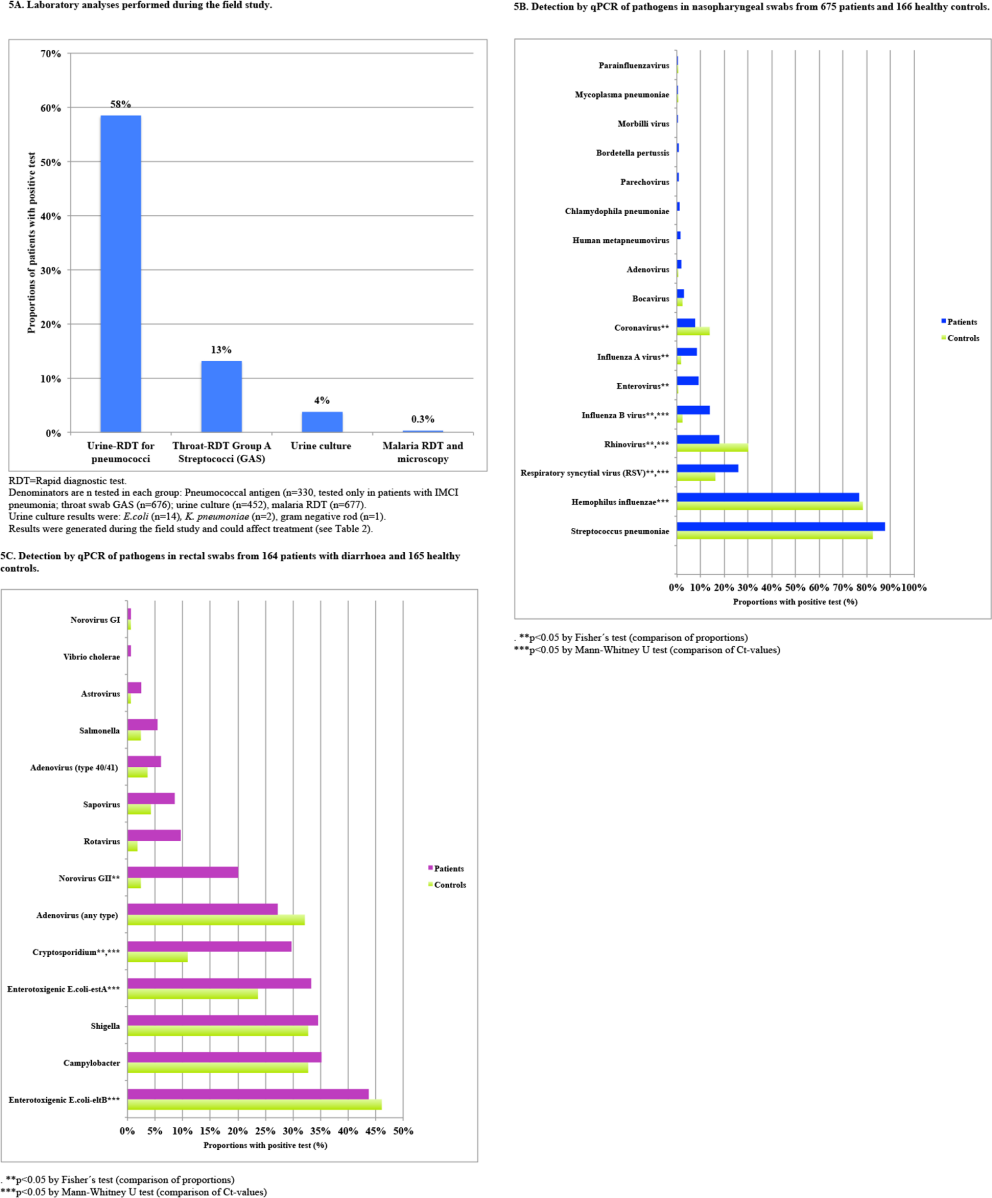
ABC. Pathogen detection in samples collected on enrolment day.

A total of 22257 analyses were conducted for detection of 36 pathogens. Crude qPCR detection rates of nasopharyngeal pathogens in patients and healthy controls as well as a comparison of Ct values between the groups are presented in Fig 5A, 5B, 5C and S2 Table. NPH qPCR detected one or more pathogens in 657/672 (98%) patients (median 3 pathogens, range: 1–7) and in 158/166 (95%) controls (median 2 pathogens, range: 1–7). Significantly higher detection rates were found in patients than controls for enterovirus, influenza A, influenza B and RSV (OR from 1.8 to 15.6, P<0.01, S1 Appendix, S1 File, S2 Table). Multiple pathogens were detected in 591 (88%) patients and 137 (83%) controls.

As previously reported [7], rectal swab qPCR detected at least one diarrheal (GE) pathogen in 153/164 (93%) patients with diarrhoea (median 2 pathogens; range 1–6) and 144/165 (87%) controls (median 2 pathogens; range 1–6) (Fig 5C). Multiple pathogens were detected by GE-qPCR in 49/164 (30%) patients and 60/165 (36%) controls. Significantly higher detection rates in patients than controls were seen for *Cryptosporidium*, norovirus genogroup II, and rotavirus (P≤0.001). *Shigella* and ETEC-estA were also identified as causes of gastroenteritis by their significantly higher pathogen load in faeces from patients as compared with controls [7].

Fig 5A shows urine culture, point-of-care and blood test results, all performed exclusively in patients. Two patients had *Plasmodium falciparum* detected by RDT, microscopy and PCR. All 83 specimens subjected to PCR-analyses for additional blood pathogens (rickettsiosis, chikungunya, dengue, Rift Valley fever and West Nile viruses) were negative. When all microbiology results (point-of-care, urine culture and PCRs) were pooled (Fig 5A,5B and 5C) a median of three pathogens (range 0–10) were observed per patient. Eight (1.2%) patients had no detectable pathogen.

### CXR-confirmed pneumonia

Out of 387 patients with IMCI-pneumonia classification, 359 underwent CXR, which confirmed pneumonia in 12% (42/342) of patients with sufficient CXR quality. Univariate and multivariate regression analysis determined CRP as the only independent variable significantly associated with CXR-confirmed pneumonia (Table 3), with CRP-levels >80mg/l corresponding to an OR of 5.9 (CI: (2.4–14.3), p<0.001). NPH-qPCR detected pneumococci in 37, RSV in 11, influenza A or B in 6, and enterovirus in 2 of the 42 patients with CXR-confirmed pneumonia.

### Final diagnoses

**Fig 6:**
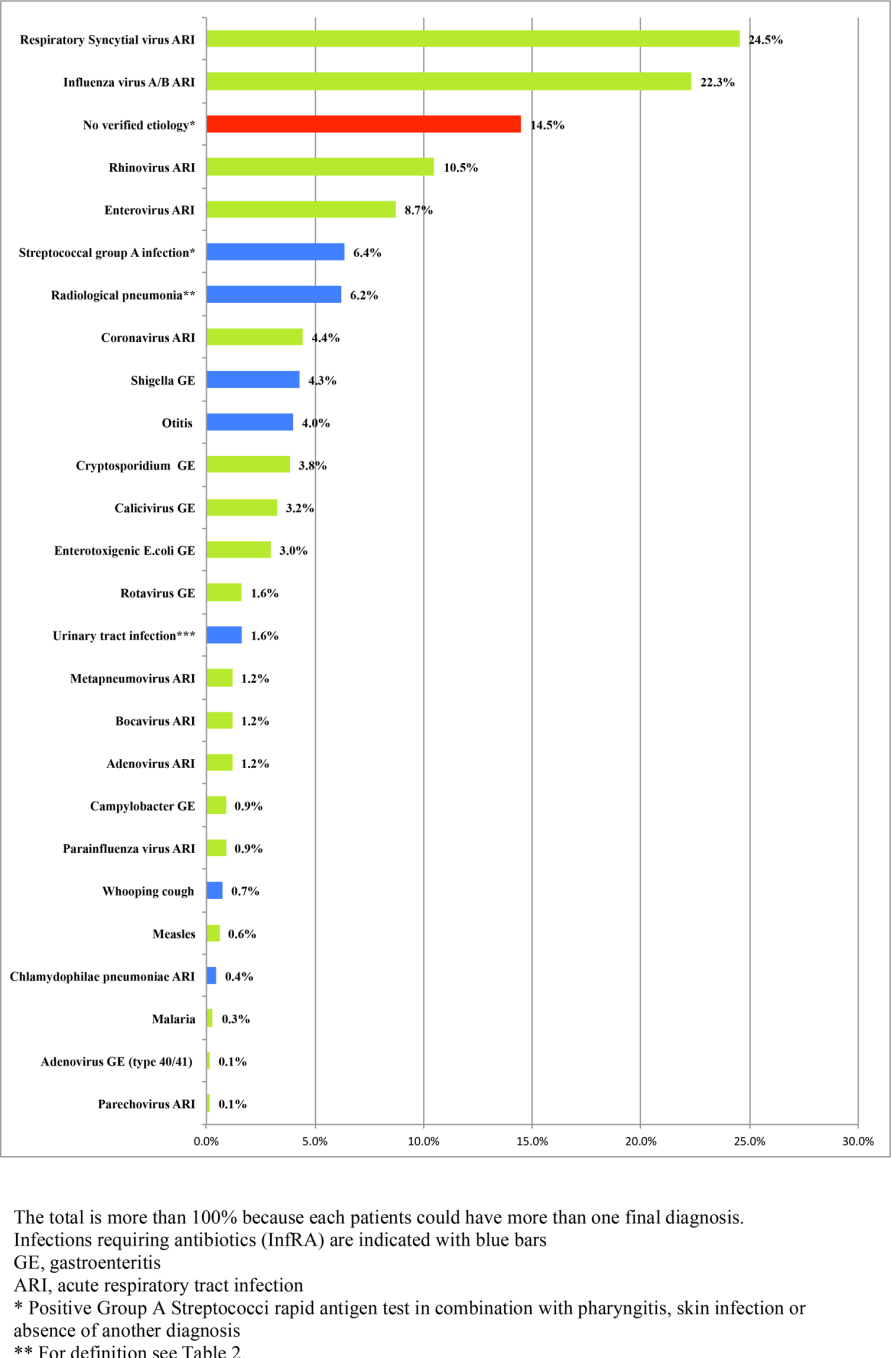
Final diagnoses in patients.

**Fig 7:**
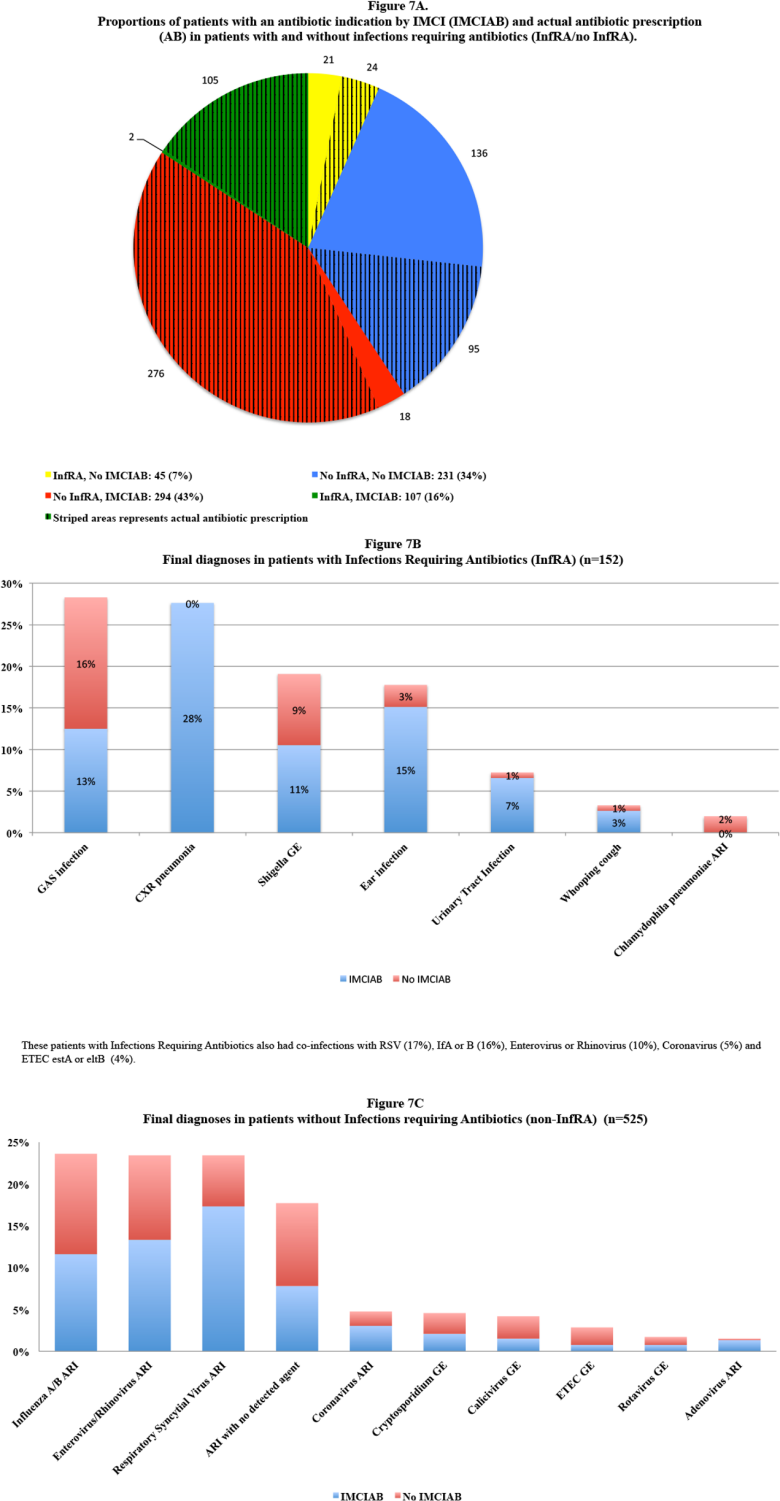
A: Proportions of patients with an antibiotic indication by IMCI (IMCIAB) and actual antibiotic prescription (AB) in patients with and without infections requiring antibiotics (InfRA/no InfRA). B: Final diagnoses in patients with Infections Requiring Antibiotics (InfRA). C: Final diagnoses in patients without Infections Requiring Antibiotics (non-InfRA).

A total of 579/677 (86%) patients were assigned a final diagnosis (Fig 6), including 160 with multiple diagnoses, resulting in 769 diagnoses in total. The most common diagnoses were viral respiratory tract infection caused by RSV, influenza B, rhinovirus, enterovirus and influenza A, whereas the most common bacterial infections were Group A streptococci (GAS), CXR-confirmed pneumonia and *Shigella* gastroenteritis (Fig 6, Fig 7B and 7C).

### Severe disease

The two deceased children, out of seven that developed signs of severe illness after enrolment (Fig 3), were assigned influenza A and enterovirus as final diagnoses. Both received antibiotics at enrolment. The remaining five children had coronavirus and rhinovirus infection (n = 2), bocavirus, GAS infection and influenza A, respectively. There was neither any difference in early fever resolution (Table 1) among patients with (39/45; 87%) or without (504/595; 85%) a serious bacterial infection (CXR-confirmed pneumonia and/or urinary tract infection [14], Table 1), nor among patients treated (392/474, 83%) or not treated (143/174, 82%) with antibiotics (p>0.05 for both).

### Antibiotic prescriptions and infections requiring antibiotics

At enrolment, 500 (74%) patients were prescribed antibiotics of whom 472 (93%) received beta lactams (Table 4). Among the 152/677 (22%) patients with infections requiring antibiotics (Fig 7A and 7B) 129 (85%) received antibiotics, the most common being GAS-infection and CXR-confirmed pneumonia. The remaining 23 that were not prescribed antibiotics recovered, although one had an episode of convulsions during follow-up. Among the 525 patients without infections requiring antibiotics (Fig 7A and 7C), 294 (56%) had IMCI indication for antibiotics, a majority of them (271/294; 92%) due to IMCI-pneumonia. Conversely, 45/152 patients (30%) with infections requiring antibiotics had no IMCI indication for antibiotics. Yet 24 of these 45 (53%) received treatment.

## Discussion

This is, to our knowledge, the first aetiology study on non-severe fever in African children that both applies a comprehensive laboratory panel and includes a healthy control group. Viral respiratory tract infections were identified as the most common fever cause. A vast majority, 98%, of patients had at least one detectable pathogen. However, some agents did not qualify as causal aetiologies (e.g. pneumococci by NPH-qPCR), and 15% of the patients could not be assigned a final diagnosis. The results, with multiple pathogens being detected in a large proportion of specimens from both patients and controls, underline the complexity of childhood infections. Also, many pathogens were detected in similar frequencies in patients and controls, as previously observed [7,8], emphasizing the need to incorporate controls in studies on causes of fever.

By comparing qPCR Ct-values (in addition to crude positivity rates) as proxy for pathogen load in patients and controls and applying multiple regression analysis to control for confounding factors, the identification of causative agents was likely improved in our study (S1 Appendix, S2 Table, Tables A and B in S1 File). Similar approaches for aetiology identification have previously been used for gastrointestinal [7,15] as well as nasopharyngeal pathogens [8,16]. Overall, the NPH-qPCR results cohered with previous studies of acute respiratory tract infections in African preschool children [17,18]. However, the observation in our study that enterovirus was a common fever cause is novel and calls for confirmatory studies that like this study distinguish between enterovirus and rhinovirus detection.

A strength of our study is that, based on all clinical and microbiology data, we retrospectively assigned individual final patient diagnoses, which allowed assessment of whether antibiotics were prescribed to those presumed to benefit from treatment. IMCI management has previously been shown to reduce inappropriate antibiotic prescription in comparison with standard fever case management [19] and a majority of the antibiotic-requiring infections in our study were indeed treated with antibiotics. Still, IMCI management resulted in substantial over-prescription of antibiotics in comparison with the final fever diagnoses retrieved based on all available clinical and laboratory data and their corresponding antibiotic requirement. Thus, among the 57% patients fulfilling the IMCI-pneumonia classification, only 12% had CXR-confirmed pneumonia. Similar results have been reported by others in both hospitalized children [20,21], and in non-severe IMCI-pneumonias [22], indicating that IMCI-pneumonia classification, based on presence of cough and rapid breathing has a low precision to identify lower antibiotic requiring respiratory tract infections. IMCI-pneumonia classification was the largest contributor to presumed unnecessary antibiotic prescription in our study. In a multiple logistic regression analysis, CRP was the only positive predictor of CXR-confirmed pneumonia. However, approximately 50% with CXR-confirmed pneumonia in our study had CRP <80 mg/L and a viral pathogen detected by NPH-qPCR, suggesting that some of the CXR-confirmed pneumonias might only have been of viral origin [23]. The IMCI guidelines have remained quite similar since its introduction, with the exception of the recent integration of malaria RDT, which has been proven useful in Zanzibar [10]. If IMCI in combination with CRP and/or other biomarkers could play a similar role by reliably confirming or excluding bacterial infections like pneumonia needs to be further studied [5]. When planning such studies on infections requiring antibiotics, the following issues should be addressed: Should severely and/or non-severely ill patients be included? How and when should the illnesses be identified (e.g point-of-care versus molecular based diagnostic techniques)? The likelihood of finding a single laboratory test/biomarker in the near future that could distinguish all infections presumed to require antibiotics from other infections is low. For instance, Fig 7B shows that GAS-infections and Shigellosis were the main infections requiring antibiotics that the IMCI algorithm did not identify. Hence, the way forward would probably be a more complex step-wise approach combining clinical signs, multiple biomarkers and point of care detection of pathogens.

The overall health outcome was satisfactory with relatively few children who developed severe disease during follow-up. One reason for this might be the ability of the clinicians to identify danger signs on enrolment. Our results concur with previous studies of IMCI management of uncomplicated childhood fever on first referral level of Pakistan and Papua New Guinea, which also reported no difference in outcome between children treated or not treated with antibiotics [24,25].

Similar distribution of fever causes as observed in our study with a predominance of viral infections and <10% serious bacterial infections (7.4% in our study) [14] have been reported in high-income countries [26]. Due to differences in methodology and inclusion criteria it is difficult to compare our findings to previous studies of fever aetiologies in African children [27–31]. A recent study from Tanzania also applied a broad microbiology test panel, and reported viral infections as the most common fever causes [32]. However, there are some important methodological differences between D'Acremont et al and our study; they included older children and, importantly, did not include a control group of asymptomatic children.

Our study has several limitations. Firstly, it was conducted during the time period, directly following the main rainy season in Zanzibar, when respiratory tract infections are known to be more frequent and some diarrheal infections like rotavirus are less frequent and might therefore not be representative for infections occurring during a whole year. Secondly, controls were only sampled for nasopharyngeal and rectal swabs, and the lack of controls for other tests might for example have resulted in over-estimation of GAS infections. Thirdly, we chose not to include blood cultures due to an assumed low yield of bacteraemias [33]. This focus on acute uncomplicated fever precludes any conclusions regarding the frequency or outcome of severe infections.

In conclusion, this study on aetiology, antibiotic treatment and outcome of non-severe febrile childhood illness in a malaria pre-elimination setting of Africa, the first using a comprehensive laboratory panel and including a healthy control group, shows the complexity of determining infectious aetiologies. The majority of fevers were caused by viral upper respiratory tract infections, similarly to children in high-income countries. A majority of asymptomatic children had potential pathogens detected by NPH-qPCR, often in a similar proportion as patients, underlining the need to include controls. The precision of IMCI to identify infections presumed to require antibiotics was low.

## Supporting Information

S1 Strobe Checklist (DOCX)

S1 Appendix (DOCX)

S1 File Table A. Pathogen detection by qPCR in nasopharyngeal and rectal swabs from patients and healthy controls. Table B. Pathogen detection by point-of care tests and microbiology in patients. (DOCX)

S1 Table Primers and probes targeting RNA or DNA from all agents detected with qPCR/PCR. (DOCX)

S2 Table Multiple logistic regression analysis of pathogens detected in nasopharyngeal swabs from patients and controls. (DOCX)

## Abbreviations

CXR: Chest X-ray
GAS: Group A Streptococci
IMCI: Integrated Management of Childhood Illness guidelines
NPH: Nasopharyngeal
qPCR: Quantitative/ Real-time Polymerase Chain Reaction
RDT: Rapid Diagnostic Test
