# Supplementary material for: Acute Uncomplicated Febrile Illness in Children Aged 2-59 months in Zanzibar – Aetiologies, Antibiotic Treatment and Outcome
Source: PLoS One. 2016 Jan 28;11(1):e0146054. doi: 10.1371/journal.pone.0146054 (PMC4731140; doi:10.1371/journal.pone.0146054)
Supplement: S1 Strobe Checklist — (DOCX) [file pone.0146054.s001.docx]

STROBE Statement—checklist of items that should be included in reports of observational studies

***Below the STROBE check list items are addressed and marked red, bold in italics.***

# Item

**Page**

**No. Recommendation No.**

**Title and abstract** 1 (*a*) Indicate the study’s design with a commonly used term in the title or the abstract ***see abstract***

(*b*) Provide in the abstract an informative and balanced summary of what was done and what was found ***see abstract***

# Introduction

Background/rationale 2 Explain the scientific background and rationale for the investigation being reported

***p5-6 line 101-115***

Objectives 3 State specific objectives, including any prespecified hypotheses

***“We therefore studied the aetiology and outcome of acute uncomplicated febrile illness in children 2-59 months seeking care at primary health care level in Zanzibar”… “We also assessed the utility of IMCI in identification of infections presumed to require antibiotics”***

# Methods

Study design 4 Present key elements of study design early in the paper

***Setting*** 5 Describe the setting, locations, and relevant dates, including periods of recruitment, exposure, follow-up, and data collection

***“This was a prospective descriptive health facility based study conducted in North A District, Zanzibar, Tanzania between April-July 2011 that followed children with acute uncomplicated febrile illness for 14 days. A healthy community control group was recruited for comparison during the same study time period.”***

***“The study district is mainly rural with approximately 100,000 inhabitants. Malaria positivity rate has declined dramatically over the past decade from approximately 40% to 1-2% in febrile patients after wide-scale deployment of malaria control interventions. Public health care is delivered through 12 primary health care units and one primary health care centre (Kivunge). In addition to first-level outpatient care, Kivunge primary health care centre has facilities for basic inpatient care and laboratory services. It was selected as study site based on its central location in the district, 24-hour service, presence of a research laboratory, and radiology equipment.“***

Participants 6 (*a*) *Cohort study*—Give the eligibility criteria, and the sources and methods of selection of participants.

Describe methods of follow-up

*Case-control study*—Give the eligibility criteria, and the sources and methods of case ascertainment and control selection. Give the rationale for the choice of cases and controls *Cross-sectional study*—Give the eligibility criteria, and the sources and methods of selection of participants

(*b*) *Cohort study*—For matched studies, give matching criteria and number of exposed and unexpo 5-6

*Case-control study*—For matched studies, give matching criteria and the number of controls per case

***Patients:***

***“Children presenting at the study site were screened for eligibility. Up to 15 patients were enrolled daily Monday-Saturday. Inclusion criteria were: age 2-59 months; acute uncomplicated fever defined as history of fever in the preceding 24 hours (information from caretaker) and/or verified fever (axillary temperature of ≥37·.5˚C by electronic thermometer); and written informed proxy consent from an accompanying caretaker. Exclusion criteria were: signs of severe disease (according to IMCI); previous study enrolment in the last 28 days; and reported inability to return for follow-up.”***

***Healthy controls:***

***“Healthy controls (hereafter referred to as “controls”), defined as children aged 2-59 months with no history of diarrhoea, cough, running nose or fever (by history and/or electronic axillary temperature <37.5°C) in the preceding ten days were recruited during the same study time period. Recruitment aimed at a representative distribution of age, sex and geography. Based on previous health facility data, eight villages in the catchment area with high attendance to the study PHCC were identified. Each study week, one of these eight villages was visited and asymptomatic children were identified through house-to-house screening. Eligible children, maximum two per household, provided NPH and rectal swabs for qPCR-analyses (Box 1).”***

Variables 7 Clearly define all outcomes, exposures, predictors, potential confounders, and effect modifiers.

Give diagnostic criteria, if applicable

***Also see Table 1AB***

***“Assessment of final diagnoses***

***Criteria for the respective final diagnoses were defined by two study investigators (paediatricians) based on all available clinical and radiology/laboratory data from day 0 (Table 1AB, Fig. 1) including the multiple regression analysis of qPCR results described further in S1-Appendix (J). Subsequently these criteria were applied on each patient. The diagnoses were categorized into three groups: 1) more probable causes of fever, 2) less probable causes of fever, and 3) “no verified aetiology”. If a more probable diagnosis was identified, all less probable diagnoses were ignored for that patient, but within one group each patient could receive more than one diagnosis.***

***Assessment of infections requiring antibiotics***

***Study definitions are outlined in Table 1A. Infections requiring antibiotics were defined retrospectively as those final diagnoses presumed to benefit from antibiotics based on WHO recommendations (***[***1***](#_ENREF_1)***). The association between these infections and both actually prescribed antibiotics and IMCI indication for antibiotics was assessed.”***

Data sources/ measurement

8* For each variable of interest, give sources of data and details of methods of assessment (measurement). Describe comparability of assessment methods if there is more than one group

***Pls see supplementary material. Methods.***

Bias 9 Describe any efforts to address potential sources of bias **4**

Study size 10 Explain how the study size was arrived at

***“This was an exploratory study, which precluded a sample size calculation. However, we estimated that a sample of 650 patients would be sufficient to obtain a representative classification of fever causes according to IMCI, and that at least 150 controls would be required to make comparisons of microbiological findings obtained by PCR analysis of nasopharyngeal and rectal specimens. “***

Continued on next page

Quantitative variables Statistical methods

# Results

1. Explain how quantitative variables were handled in the analyses. If applicable, describe which groupings were chosen and why

1. (*a*) Describe all statistical methods, including those used to control for **confounding 6,**

1. Describe any methods used to examine subgroups and interactions
2. Explain how missing data were addressed
3. *Cohort study*—If applicable, explain how loss to follow-up was addressed

*Case-control study*—If applicable, explain how matching of cases and controls was addressed *Cross-sectional study*—If applicable, describe analytical methods taking account of sampling strategy

***See also Table 1, Table 3 and S3-Table***

***Missing data: Table 1AB, Table 2, Figure 2.***

***“On every follow-up visit a study clinician performed clinical reassessment including axillary temperature. If a child did not return for a scheduled follow-up, it was actively traced at home. Any patient with symptoms/signs of severe disease, predefined abnormal laboratory values or withdrawal of consent during the 14-day follow-up was discontinued from the study (Fig. 2).”***

***“Data were double entered in CSPro, validated and exported to STATA® 12 where all statistical analyses were performed. Frequencies, proportions and odds ratios (ORs) were calculated with 95% confidence intervals (CI). P-values <0.05 were considered statistically significant. Fisher’s exact test and exact binomial test was used for binary data and proportions, two-sample t-test for comparisons of means, and Mann-Whitney-U test for median comparisons. In a univariate and multivariate logistic regression we assessed the association between CXR-confirmed pneumonia and the continuous and binary variables outlined in Table 3. WBC (>20x 10^9/L) and CRP cut-off values (<20 and >80 mg/L), were chosen to concur with published literature”***

Participants 13* (a) Report numbers of individuals at each stage of study—eg numbers potentially eligible, examined for eligibility, confirmed eligible, included in the study, completing follow-up, and analysed

- 1. Give reasons for non-participation at each stage
  2. Consider use of a flow diagram

***Please see Fig 2. Flow of patients through the study***

Descriptive data 14* (a) Give characteristics of study participants (eg demographic, clinical, social) and information on exposures and potential confounders**.**

1. Indicate number of participants with missing data for each variable of interest
2. *Cohort study*—Summarise follow-up time (eg, average and total amount)

***Please see Table 2, Fig 2 and Fig 4ABC.***

Outcome data 15* *Cohort study*—Report numbers of outcome events or summary measures over **time**

*Case-control study—*Report numbers in each exposure category, or summary measures of exposure

*Cross-sectional study—*Report numbers of outcome events or summary measures

Main results 16 (*a*) Give unadjusted estimates and, if applicable, confounder-adjusted estimates and their precision (eg, 95% confidence interval). Make clear which confounders were adjusted for and why they were included

1. Report category boundaries when continuous variables were categorized
2. If relevant, consider translating estimates of relative risk into absolute risk for a meaningful time period

***Please see qll results.***

Continued on next page

# Discussion

Key results 18 Summarise key results with reference to study objectives

***“This is, to our knowledge, the first aetiology study on non-severe fever in African children that both applies a comprehensive laboratory panel and includes a healthy control group. Viral respiratory tract infections were identified as the most common fever cause.***

***A vast majority, 98%, of patients had at least one detectable pathogen. However, some agents did not qualify as causal aetiologies (e.g. pneumococci by NPH-qPCR), and 15% of the patients could not be assigned a final diagnosis. The results, with multiple pathogens being detected in a large proportion of specimens from both patients and controls, underline the complexity of childhood infections. Also, many pathogens were detected in similar frequencies in patients and controls, as previously observed emphasizing the need to incorporate controls in studies on causes of fever. “***

Limitations 19 Discuss limitations of the study, taking into account sources of potential bias or imprecision. Discuss both direction and magnitude of any potential bias

***“Our study has several limitations. Firstly, it was conducted during the time period, directly following the main rainy season in Zanzibar, when respiratory tract infections are known to be more frequent and some diarrheal infections like rotavirus are less frequent and might therefore not be representative for infections occurring during a whole year. Secondly, controls were only sampled for nasopharyngeal and rectal swabs, and the lack of controls for other tests might for example have resulted in over-estimation of GAS infections. Thirdly, we chose not to include blood cultures due to an assumed low yield of bacteraemias. This focus on acute uncomplicated fever precludes any conclusions regarding the frequency or outcome of severe infections. “***

Interpretation 20 Give a cautious overall interpretation of results considering objectives, limitations, multiplicity of analyses, results from similar studies, and other relevant evidence

***“In conclusion, this study on aetiology, antibiotic treatment and outcome of non-severe febrile childhood illness in a malaria pre-elimination setting of Africa, the first using a comprehensive laboratory panel and including a healthy control group, shows the complexity of determining infectious aetiologies. The majority of fevers were caused by viral upper respiratory tract infections, similarly to children in high-income countries. A majority of asymptomatic children had potential pathogens detected by NPH-qPCR, often in a similar proportion as patients, underlining the need to include controls. The precision of IMCI to identify infections presumed to require antibiotics was low. “***

Generalisability 21 Discuss the generalisability (external validity) of the study results

“***Similar distribution of fever causes as observed in our study with a predominance of viral infections and <10% serious bacterial infections (7.4% in our study) (***[***2***](#_ENREF_2)***) have been reported in high-income countries. Due to differences in methodology and inclusion criteria it is difficult to compare our findings to previous studies of fever aetiologies in African children. A recent study from Tanzania also applied a broad microbiology test panel, and reported viral infections as the most common fever causes. However, there are some important methodological differences between D'Acremont et al and our study; they included older children and, importantly, did not include a control group of asymptomatic children.”***

# Other information

Funding 22 Give the source of funding and the role of the funders for the present study and, if applicable, for the original study on which the present article is based,

***Please see submission***

*Give information separately for cases and controls in case-control studies and, if applicable, for exposed and unexposed groups in cohort and cross-sectional studies.

**Note:** An Explanation and Elaboration article discusses each checklist item and gives methodological background and published examples of transparent reporting. The STROBE checklist is best used in conjunction with this article (freely available on the Web sites of PLoS Medicine at [http://www.plosmedicine.org/,](http://www.plosmedicine.org/) Annals of Internal Medicine at [http://www.annals.org/,](http://www.annals.org/) and Epidemiology at [http://www.epidem.com/).](http://www.epidem.com/)) Information on the STROBE Initiative is available at [www.strobe-statement.org.](http://www.strobe-statement.org/)
