# Supplementary material for: Acute Uncomplicated Febrile Illness in Children Aged 2-59 months in Zanzibar – Aetiologies, Antibiotic Treatment and Outcome
Source: PLoS One. 2016 Jan 28;11(1):e0146054. doi: 10.1371/journal.pone.0146054 (PMC4731140; doi:10.1371/journal.pone.0146054)
Supplement: S1 Appendix — (DOCX) [file pone.0146054.s002.docx]

**Appendix**

### Methods

### A. Logistics and analysis of laboratory investigations

All laboratory specimens were collected at the study site according to the manufacturers’ instructions and study specific Standard Operating Procedures (SOP) for collection and storage of specimens. The laboratory analyses of the samples were conducted on three different levels, on the study site, in the Zanzibar microbiology department (Mnazi Mmoja Hospital), in Sweden (University of Gothenburg or Karolinska Institutet) or in France (Aix Marseille Université) (Figure 1- main manuscript). All study staff handling laboratory samples were trained on a safe and proper sample collection and were further supervised by the designated study coordinator. After completion of the field trial, all samples collected for molecular analysis were transported in a controlled environment to Sweden by air either as dried blood spots (DBS) for malaria PCR or on dry ice (for rectal and nasopharyngeal swabs) or in closed preservation agar (for urine cultures).

**B. RDT Malaria**

We used the same RDT device as deployed by Zanzibar Malaria Control Programme by the time of trial. (SD bioline-Pf/Pan® detecting *P. falciparum* specific HRP2 antigen and pLDH-Pan Plasmodium specific Plasmodium lactate dehydrogenase). The tests were performed and interpreted according to the manufacturer’s instructions.

**C. Malaria microscopy**

Slides were stained with 5% Giemsa for 30 minutes and examined under oil immersion (x100 magnification) by two independent and experienced microscopists blinded to both the RDT and each other’s microscopy result. Asexual parasite densities were calculated against 200 white blood cells (WBC), assuming 8,000 WBC per microliter of blood. A slide was declared negative if no parasites were found after examining 100 high power microscopy fields.

**D. Dried blood spots (DBS)**

The blood samples (approximately 100μL) collected on filter papers (Whatmann® 3MM) were dried thoroughly, put in individual zipped plastic bags containing desiccant and stored in room temperature (<25°C) in Zanzibar until completion of the study and then transported to Sweden for molecular analyses as shown below, whereafter if not processed it was stored at <-20°C. See S1-Table for PCR primers and probes.

*a. Malaria detection by PCR:* The DBS were screened for human plasmodial infection with cytb-nested PCR as described by Hsiang et al ([1](#_ENREF_1)). Briefly, every 9 samples were pooled and subjected to Chelex DNA extraction and cytb-nested PCR. Samples from positive pools underwent individual DNA extraction and cytb-nested PCR was carried out. Positive PCR products were further analyzed by RFLP assay for species identification.

b*. Rickettsia spp. detection. Quantitative real-time reverse transcription PCR (qRT-PCR)* was performed by using a *Rickettsia* genus-specific qRT-PCR targeting the *gltA* gene of Spotted fever group *Rickettsia spp*, , an *R. felis*-specific qRT-PCR targeting the *bioB* and *orfB* genes.. ([2](#_ENREF_2)). qRT-PCR to detect Typhus group *Rickettsia* spp. targeted the hypothetical protein encoding gene ([3](#_ENREF_3)).

c. *Detection of dengue virus (DENV), West Nile virus (WNV), Rift Valley fever virus (RVFV), and chikungunya virus (CHIKV by PCR:* RNA was extracted from 84 DBS using Qiagen RNeasy Mini kit (Alameda, CA, USA) according to the manufactural protocol. Five μL RNA was used in each assay. The RNA samples were initially screened by a beta actin PCR in order to control the quality of the RNA (Beta actin Life Technologies, art NO 4333762). The dengue PCR is a semi-nested PCR published by Harris et al ([4](#_ENREF_4)). For the detection of RVFV, WNV, and CHIKV in house real-time PCRs were used. One additional real-time PCR was used for the detection of WNV ([5](#_ENREF_5)).

**E. C-reactive protein (CRP) (whole blood)**

We used a NycoCard® assay (Medinor, Lidingö, Sweden) according to the manufacturer’s instructions including quality control. 5 µL of capillary blood was diluted and 50 µL of diluted samples were added to a reaction device, followed by the addition of one drop of conjugate after 30 s one drop of washing solution. After 2 minutes the result was identified using NycoCard® Reader II. The test result were one of the following <8, >200mg/l or in the measuring range 8-200mg/l.

**F. Complete blood count (CBC)**

We used Sysmex® KX-21 according to manufacturer’s instructions to analyse complete blood count in all patients. Patients were discontinued from the study due to abnormal laboratory values (see Table 1).

**G. RDT- Group A Streptococci (GAS) (throat swab)**

We used rapid qualitative antigen detection of GAS from a throat swab with results within 15 minutes, all according to manufacturer’s instructions (QuickVue® Dipstick Strep A test). Very faint bands at the test line position were to be assessed as a positive test result. Band intensity was not recorded.

**H. Gastrointestinal panel (rectal swab)**

The rectal swab collection, storage, transport and further molecular analyses (real-time PCR technique) have previously been published ([6](#_ENREF_6))

**I. Respiratory panel (nasopharyngeal swabs and enterovirus/rhinovirus sequencing)**

The swabs (Copan Regular Flocked Swab 502CS01, Copan Italia Spa, Brescia, Italy) were sampled in a standardised manner from the nasopharynx of the child. Directly after sampling, the swab was placed into a sterile vial containing 1 ml sterile NaCl 0.9% and put in a vaccine carrier with a controlled temperature of 2-8˚C. Within 2 hours after sampling the liquid content was transferred to a micro-tube using a disposable transfer pipette and stored in a controlled temperature of –70˚C. After completion of the field trial all samples were transported to Sweden on dry ice for molecular analyses. The technique used for the nasopharyngeal samples, qPCR based on automated specimen extraction and multiplex amplification has been described before([7](#_ENREF_7)). S1-Table shows primers and probes for the NPH qPCR. When both enterovirus and rhinoviruses were positive and there was a >10 cycles difference in Ct values, the result was in favour of the pathogen with the lowest Ct value. Otherwise sequencing was performed for species identification ([8](#_ENREF_8)).

## J. Identification of Ct value cut-off, likely cause of disease by multiple regression analysis

After completion of the field study, nasopharyngeal and rectal swabs from patients and healthy controls were analysed by qPCR. The results were recorded as detection (yes or no) and for reactive samples also as the so-called Ct (threshold cycle) value, a parameter that is negatively proportional to the logarithm of the target concentration. For some agents, a Ct-value cut off was identified that optimally distinguished between patients and healthy controls as reported for gastrointestinal pathogens ([6](#_ENREF_6)), and as follows for respiratory pathogens.

Ct values from nasopharyngeal qPCR were first compared in a logistic regression, first univariate and then multivariate analysis with patient/control as dependent variable and test positivity (binary), age (continuous), and sex (binary) as independent variables. Factors not independently associated with symptoms were omitted in a step-wise manner. Agents independently associated with disease with a difference in crude detection rate between patients and controls were classified as more likely causes of disease.

**K. Urine sample collection (clean catch urine)**

A clean catch urine specimen was collected in a sterile, dry, wide-necked and leak-proof container after giving instructions to the caretaker to clean the external genitalia of the child and then wait for the child to pass urine.

**a. Rapid Diagnostic Test - pneumococci (urine)**

We used BINAX NOW® for rapid detection of pneumococci antigens in urine with results within 15 minutes, all according to manufacturer’s instructions. Very faint bands at the test line position were to be assessed as a positive test result. Band intensity was not recorded. The pneumococcal antigen was processed within 15 minutes after sample collection.

**b. Urine dipstick**

We used CombiScreen® (7 SYS PLUS) for semi-quantitative biochemical detection of protein, nitrite, erythrocytes, and leukocyte esterase according to the manufacturer’s instructions. The test result did not affect the management of the patient.

**c. Urine culture**

Study specific SOPs for urine culture collection, storage, transport, processing, quality control and results reporting were developed in collaboration with Mnazi Mmoja Microbiology Laboratory in Zanzibar. The procedures followed WHO standards ([9](#_ENREF_9), [10](#_ENREF_10))

Cysteine lactose electrolyte deficient (CLED) agar was prepared as specified by manufacturers and dispensed unto petri dishes. The collected sample for urine culture was stored in a refrigerator (controlled temperature; 2-8˚C) for not more than 24 hours (usually <8 hours) before inoculation. During the daily transport, one-hour-drive from the study laboratory to Mnazi Mmoja microbiology department, the specimens were sent in a portable vaccine carrier with ice-blocks in a controlled temperature, 2-8˚C.

After inoculation with a sterile plastic wire loop the petri dish was placed in an incubator 35-37˚C, growth was controlled after 16-24 hours.

If a result showing significant growth of one bacteria (≥10^7^cfu/L), Gram-stain was used to differ between gram-positive and Gram-negative bacteria, and additional tests were then performed for identification. Staphylococci were identified using catalase, DNAse, and novobiocin tests, streptococci were decided to Lancefield-group using Streptex®, *pseudomonas* *spp* were identified using oxidase-test and *enterobacteriaceae* were identified and decided to species level using BioMerieux API® biotyping system.

As an external quality control, re-culturing of urine culture colonies was done in the Department of Microbiology, University of Gothenburg, to verify the species of pathogen in urine and its antibiotic susceptibility pattern. Fresh colonies from positive urine cultures (i.e. with significant growth on the plates) were collected in a sterile manner with a plastic loop and placed in preservation agar, which served as a transport medium for the bacterial colonies. In case of conflict, the Gothenburg result was decisive and recorded as the final result.

#### L. Chest X-ray procedure

Analog chest X-rays (CXR) were performed on-site using an anterior-posterior view according to WHO recommended standards and within three days interpreted by a radiologist in the main referral hospital on the island (Mnazi Mmoja hospital) as either primary endpoint pneumonia/consolidation, other infiltrate, normal, or un-interpretable, i.e. not of sufficient quality ([11](#_ENREF_11)). In case of any significant severe pathology observed on the CXR, the patient was discontinued from the study and referred to a designated paediatric specialist in Zanzibar main referral hospital. After field study completion all CXR-films were digitalised according to WHO standards ([11](#_ENREF_11)) and interpreted by an experienced paediatric radiologist in Sweden. All discordant CXR results regarding “primary endpoint pneumonia” or “no primary endpoint pneumonia ” between the two readers were subjected a third and decisive interpretation by an experienced radiologist in Sweden blinded to the previous reports.

**Results**

Crude qPCR detection rates of nasopharyngeal pathogens in patients and healthy controls as well as a comparison of Ct values between the groups are presented in S1-File.. Nasopharyngeal qPCR detection rates were higher in patients than controls for enterovirus, influenza A and B, and RSV whereas median Ct-values for RSV and influenza B were significantly lower in patients than controls. S2-Table shows those pathogens that multivariate logistic regression analysis classified as more likely causes of disease. However in contrast to rectal swab qPCR ([6](#_ENREF_6)), a cut-off could not be identified for any nasopharyngeal pathogen.

1. Hsiang MS, Lin M, Dokomajilar C, Kemere J, Pilcher CD, Dorsey G, et al. PCR-based pooling of dried blood spots for detection of malaria parasites: optimization and application to a cohort of Ugandan children. Journal of clinical microbiology. 2010;48(10):3539-43. Epub 2010/08/06.

2. Mediannikov O, Socolovschi C, Edouard S, Fenollar F, Mouffok N, Bassene H, et al. Common epidemiology of Rickettsia felis infection and malaria, Africa. Emerging infectious diseases. 2013;19(11):1775-83. Epub 2013/11/06.

3. Walter G, Botelho-Nevers E, Socolovschi C, Raoult D, Parola P. Murine typhus in returned travelers: a report of thirty-two cases. The American journal of tropical medicine and hygiene. 2012;86(6):1049-53. Epub 2012/06/06.

4. Harris E, Roberts TG, Smith L, Selle J, Kramer LD, Valle S, et al. Typing of dengue viruses in clinical specimens and mosquitoes by single-tube multiplex reverse transcriptase PCR. Journal of clinical microbiology. 1998;36(9):2634-9. Epub 1998/08/15.

5. Huang C, Slater B, Rudd R, Parchuri N, Hull R, Dupuis M, et al. First Isolation of West Nile virus from a patient with encephalitis in the United States. Emerging infectious diseases. 2002;8(12):1367-71. Epub 2002/12/25.

6. Elfving K, Andersson M, Msellem MI, Welinder-Olsson C, Petzold M, Bjorkman A, et al. Real-time PCR threshold cycle (Ct) cut-offs help to identify agents causing acute childhood diarrhea in Zanzibar. Journal of clinical microbiology. 2014. Epub 2014/01/10.

7. Andersson ME, Olofsson S, Lindh M. Comparison of the FilmArray assay and in-house real-time PCR for detection of respiratory infection. Scandinavian journal of infectious diseases. 2014:1-5. Epub 2014/10/08.

8. Sansone M, Andersson M, Brittain-Long R, Andersson LM, Olofsson S, Westin J, et al. Rhinovirus infections in western Sweden: a four-year molecular epidemiology study comparing local and globally appearing types. European journal of clinical microbiology & infectious diseases : official publication of the European Society of Clinical Microbiology. 2013;32(7):947-54. Epub 2013/02/26.

9. Cheesbrough M. District Laboratory Practice in Tropical countries. Cambridge: Co-published by the press syndicate of The University of Cambridge andTropical Health Technology; 2001.

10. Vandepitte J. Basic laboratory procedures in clinical bacteriology. 2nd ed. Geneva: World Health Organization; 2003.

11. Cherian T, Mulholland EK, Carlin JB, Ostensen H, Amin R, de Campo M, et al. Standardized interpretation of paediatric chest radiographs for the diagnosis of pneumonia in epidemiological studies. Bull World Health Organ. 2005;83(5):353-9. Epub 2005/06/25.
