# Supplementary material for: Acute Uncomplicated Febrile Illness in Children Aged 2-59 months in Zanzibar – Aetiologies, Antibiotic Treatment and Outcome
Source: PLoS One. 2016 Jan 28;11(1):e0146054. doi: 10.1371/journal.pone.0146054 (PMC4731140; doi:10.1371/journal.pone.0146054)
Supplement: S1 File — (DOCX) [file pone.0146054.s003.docx]

**S1-File**

**Table A Pathogen detection by qPCR in nasopharyngeal and rectal swabs from patients and healthy controls**

**Table B Pathogen detection by point-of care tests and microbiology in patients.**

| **Pathogen** | **Patients n positive (%)** | **Controls  n positive (%)** | **Unadjusted OR** | **P^a^** | **Ct value median (patients/controls)** | **P^b^** |
| --- | --- | --- | --- | --- | --- | --- |
| **Table A**  ***Nasopharyngeal swab qPCR (n sampled)*** | **672** | **166** |  |  |  |  |
| **Viruses** |  |  |  |  |  |  |
| Adenovirus | 12 (1.8%) | 1 (0.6%) | 3 | 0.48 | 29.5/36.6 | 0.42 |
| Bocavirus | 20 (3,0%) | 4 (2.4%) | 1.2 | 1.0 | 36.7/38.3 | 0.19 |
| Coronavirus | 52 (7.7%) | 23 (14%) | 0.5 | 0.02 | 31.9/31.7 | 0.33 |
| Enterovirus | 58 (8.6%) | 1 (0.6%) | 15.6 | <0.0001 | 36.0 /35.4 | 0.81 |
| Metapneumovirus | 11 (1.6%) | 0 (0%) | . | 0.13 | 28.6/0 | . |
| Influenza B virus | 94 (14%) | 4 (2.4%) | 6.6 | <0.0001 | 27.8/34.7 | 0.0083 |
| Influenza A virus | 58 (8.6%) | 3 (1.8%) | 5.1 | 0.001 | 27.3/39.0 | 0.35 |
| Morbillivirus | 5 (0.7%) | 0 (0%) | . | 0.6 | 37.8/0 | . |
| Parechovirus | 5 (0.7%) | 0 (0%) | . | 0.6 | 32.6/0 | . |
| Parainfluenza virus | 10 (1.5%) | 1 (0.6%) | 2.5 | 0.7 | 24.9/23.8 | 0.75 |
| Respiratory syncytial virus (RSV) | 174 (26%) | 27 (16%) | 1.8 | 0.008 | 26.8/33.7 | 0.0025 |
| Rhinovirus | 105 (16%) | 48 (29%) | 0.5 | <0.0001 | 30.7/28.7 | 0.022 |
| **Bacteria** |  |  |  |  |  |  |
| *Bordetella pertussis* | 5 (0.7%) | 0 (0%) | . | 0.59 | 30.2/0 | . |
| *Chlamydophila pneumoniae* | 8 (1.2%) | 0 (0%) | . | 0.37 | 40.4/0 | . |
| *Hemophilus influenzae* | 515 (77%) | 130 (78%) | 0.93 | 0.76 | 30.2/29.4 | 0.023 |
| *Mycoplasma pneumoniae* | 2 (0.3%) | 1 (0.6%) | 0.5 | 0.49 | 38.2/37.9 | 1 |
| *Streptococcus pneumoniae* | 587 (87%) | 137 (83%) | 1.5 | 0.128 | 27.3/26.9 | 0.08 |
| ***Rectal swab qPCR (n sampled)*** | **164** | **165** |  |  |  |  |
| **Viruses** |  |  |  |  |  |  |
| Adenovirus (any) | 45 (27%) | 53 (32%) | 0.79 | 0.4 | 38.2/39.3 | 0.05 |
| Adenovirus 40/41 | 10 (6.1%) | 6 (3.6%) | 1.71 | 0.44 | 36.6./35.0 | 0.66 |
| Astrovirus | 4 (2.4%) | 1 (0.6%) | 4.07 | 0.37 | 19.9/31.5 |  |
| Norovirus GI | 1 (0.6%) | 1 (0.6%) | 1 |  |  |  |
| Norovirus GII | 33 (20%) | 4 (2.4%) | 10.1 | <0.0001 | 25.1/26.9 | 0.28 |
| Rotavirus | 16 (9.8%) | 3 (1.8%) | 5.8 | 0.003 | 24.4/26.0 | 0.5 |
| Sapovirus | 13 (7.9%) | 7 (4.2%) | 2.09 | 0.18 | 25.6/28.3 | 0.5 |
| **Bacteria** |  |  |  |  |  |  |
| *Campylobacter* | 58 (35%) | 54 (33%) | 1.11 | 0.73 | 31.8/33.3 | 0.12 |
| *Vibrio cholerae* | 1 (0.6%) | 0 (0%) |  | - |  |  |
| ETEC*-eltB* | 71 (43%) | 76 (46%) | 0.91 | 0.74 | 31.3/34.6 | 0.002 |
| ETEC*-estA* | 54 (33%) | 39 (24%) | 1.62 | 0.07 | 32.6/37.3 | 0.0001 |
| *Salmonella* | 9 (5.5%) | 4 (2.5%) | 2.32 | 0.26 | 42.2/40.6 | 0.22 |
| *Shigella* | 56 (34%) | 54 (33%) | 1.08 | 0.82 | 29.2/34.5 | <0.0001 |
| *Yersinia* | 0 (0%) | 0 (0%) |  |  |  |  |
| **Protozoa** |  |  |  |  |  |  |
| *Cryptosporidium* | 49 (30%) | 18 (11%) | 3.45 | <0.0001 | 32.1/36.8 | 0.0009 |
| **Table B**  ***Throat swab (n sampled)*** | ***676*** |  |  |  |  |  |
| Rapid antigen test Group A Streptococci | 89 (13%) |  |  |  |  |  |
| ***Clean catch urine*** |  |  |  |  |  |  |
| *Streptococcus pneumoniae* urinary antigen positive (n sampled=330) | 193 (58%) |  |  |  |  |  |
| ***Urine culture (n sampled)*** | 452 |  |  |  |  |  |
| No growth | 365 (81%) |  |  |  |  |  |
| Mixed growth/not significant | 71 (16%) |  |  |  |  |  |
| Significant growth of one urinary pathogen | 16 (3.5%) |  |  |  |  |  |
| *Escherichia coli* | 14 |  |  |  |  |  |
| *Klebsiella pneumoniae* | 2 |  |  |  |  |  |
| *Gram negative rod* | 1 |  |  |  |  |  |
| ***Capillary blood (n sampled)*** | ***677*** |  |  |  |  |  |
| Rapid Diagnostic Test, *Plasmodium falciparum* positive | 2 (0.3%) |  |  |  |  |  |
| Blood smear, *Plasmodium falciparum* positive | 2 (0.3%) |  |  |  |  |  |
| Malaria PCR, *Plasmodium. falciparum* positive | 2 (0.3%) |  |  |  |  |  |
| ***Multiple pathogens positive per patient, all tests.*** |  |  |  |  |  |  |
| **0** | 8 (1.2) |  |  |  |  |  |
| **1** | 41 (6.1) |  |  |  |  |  |
| **2** | 134 (20) |  |  |  |  |  |
| **3** | 263 (39) |  |  |  |  |  |
| **4** | 130 (20) |  |  |  |  |  |
| **5** | 36 (5.3) |  |  |  |  |  |
| **6** | 34 (5.0) |  |  |  |  |  |
| **7** | 20 (3.0) |  |  |  |  |  |
| **8** | 6 (0.9) |  |  |  |  |  |
| **9** | 4 (0.6) |  |  |  |  |  |
| **10** | 1 (0.1) |  |  |  |  |  |

ETEC: Enterotoxigenic *Escherichia Coli*

OR, odds ratio

RDT: Rapid Diagnostic Test

The rectal swab qPCR results have already been published (Elfving et al, JCM, 2014)

^a^ Fisher’s exact test.

^b^ Mann-Whitney U test displayed for agents with a total of >10 positives samples.
