# Supplementary material for: Acute Uncomplicated Febrile Illness in Children Aged 2-59 months in Zanzibar – Aetiologies, Antibiotic Treatment and Outcome
Source: PLoS One. 2016 Jan 28;11(1):e0146054. doi: 10.1371/journal.pone.0146054 (PMC4731140; doi:10.1371/journal.pone.0146054)
Supplement: S1 Table — (DOCX) [file pone.0146054.s004.docx]

|  |  |  |  |
| --- | --- | --- | --- |
| **S1-Table. Primers and probes targeting RNA or DNA from all agents detected with qPCR/PCR** | | |  |
|  | **Forward primer** | **Reverse primer** | **Probe** |
| **Nasopharyngeal swab qPCR** |  |  |  |
| **Viruses** |  |  |  |
| Adenovirus | GCCACGGTGGGGTTTCTAAACTT | GCCCCAGTGGTCTTACATGCACATC | HEX-TGCACCAGACCCGGGCTCAGGTACTCCGA-BHQ1 |
| Bocavirus | CGGGCTCATATCATCAGGAAC | ATCACTTGGTCTGAGGTCTTCGA | FAM-CAATCAGCCACCTATC-MGB |
| Coronavirus 229E | CAGTCAAATGGGCTGATGCA | AAAGGGCTATAAAGAGAATAAGGTATTCT | FAM-CCTGACGACCACGTTGT-MGB |
| Coronavirus OC43 | CGATGAGGCTATTCCGACTAGGT | CCTTCCTGAGCCTTCAATATAGTAACC | NED-CCTGGCACGGTACTC-MGB |
| Coronavirus HKU1 | AAATGTGATCGTGCTATGCCAA | CTTAACATAATAGCAACCGCCACA | VIC-CCTTGCGAATGAATG-MGB |
| Coronavirus NL63 | ACGTACTTCTATTATGAAGCATGATATTAA | AGCAGATCTAATGTTATACTTAAAACTACG | NED-CCAAGGCTCCTAAACG-MGB |
| Enterovirus | ?GGTGYGAAGAGYCTATTGAGCTA | GGACACCCAAAGTAGTCGGTTC | VIC-TCCGGCCCCTGAATG-MGB |
| Metapneumovirus | ATGTCTCTTCAAGGGATTCACCT | AMAGYGTTATTTCTTGTTGCAATGATGA | FAM-CATGCTATATTAAAAGAGTCTCARTAC-MGB |
| Influenza A virus | AAGACCAATCCTGTCACCTCTGA | CAAAGCGTCTACGCTGCAGTCC | FAM-TTTGTGTTCACGCTCACCGT-MGB |
| Influenza B virus | AAATACGGTGGATTAAATAAAAGCAA | CCAGCAATAGCTCCGAAGAAA | VIC-CCATAGGAAATTGCC-MGB |
| Morbilli virus | CGTTGACCCTGACGTTAGCA | GCGAAGGTAAGGCCAGATTG | FAM-TGTCCTCAGTAGTATGCATTGCAA-BHQ1 |
| Parechovirus | GATGCCCAGAAGGTAC | TTTGGCCCACTAGACGTT | NED-CCCAGATCAGATCCA-MGB |
| Parainfluenza virus 1 | CAACAGGAAATCATGTTCTGTAATAGC | TCACAGTGGGCAAGGAGCA | NED-CTGGTAACCCCTTGTTCCTG-MGB |
| Parainfluenza virus 2 | GCATTTCCAATCTTCAGGACTATGA | ACCTCCTGGTATAGCAGTGACTGAAC | JOE-CCATTTACCTAAGTGATGGAATCAATCGCAAA-BHQ1 |
| Parainfluenza virus 3 | AAATGATCTGATTTATGCTTATACCTC | TCAGGTACCAAGTCTGAGTTTACA | FAM-CGAGGTTGYCAGGATATAGGAAAATCA-BHQ1 |
| Respiratory syncytial virus (RSV) | GCAAATATGGAAACATACGTGAACA | GCACCCATATTGTWAGTGATGCA | FAM-CTTCACGAAGGCTCCACATACACAGCWG-BHQ1 |
| Rhinovirus | GGTGTGAAGAGCCSCRTGTGCT | GCAGGGTTTRGGTTAGCCRCATT | VIC-TCCGGCCCCTGAATG-MGB |
|  | GGTGTGAAGACTCGCATGTGCT |  |  |
|  | GGGTGYGAAGAGYCTANTGTGCT |  |  |
| **Bacteria** |  |  |  |
| *Bordetella pertussis* | CGGATGAACACCCATAAGCAT | CGATCAATTGCTGGACCATTT | JOE-CCCGATTGACCTTCCTACGTCGACTC-BQ1 |
| *Chlamydia pneumoniae* | CAAGGGCTATAAAGGCGTTGCT | ATGGTCGCAGACTTTGTTCCA | NED-CCCCTTGCCAACAGA-MGB |
| *Hemophilus influenzae* | CTAACAACGATGCTGCAGGCA | GTGTTATAACGTTGTTGAAGATCAGC | NED-ATGGTGCTGCTCAAA-MGB |
| *Mycoplasma pneumoniae* | GGAATCCCAATGCACAAGAACA | GCTTTGGTCAACACATCAACCTT | NED-CAAACCCAGCCTTCA-MGB |
| *Streptococcus pneumoniae* | TTTCTGGATAGAGGGAGTATCCGA | TTACCAACCTACTCATCTTCTCACCA | FAM-CAAAGTTAATACCGCCCTC-MGB |
| **Rectal swab qPCR** |  |  |  |
| **Viruses** |  |  |  |
| Norovirus GG2 | TGGAYTTTTAYGTGCCCAG | CGACGCCATCTTCATTCAC | VIC-AGCCAGATTGCGATCGCCC-TAMRA |
| Rotavirus | AACCATCTACACATGACCCTCTATGA | GGTCACATAACGCCCCTATAGC | FAM-CAATAGTTAAAAGCTAACACTGTCAAA-MGB |
| Astrovirus | GACTGCWAAGCAGCTTCGTGA | GCTAGCCATCACACTTCTTTGGTCCT | FAM-TCACAGAAGAGCAACTCCATCGCATTTGBQ1 |
| Sapovirus | TTGGCCCTCGCCACCTAC | CCCTCCATYTCAAACACTA | VIC-CCRCCTATRAACCA-MGB |
|  | GAYCASGCTCTCGCYACCTAC |  |  |
| Norovirus GG1 | TGGCAGGCCATGTTCCGCT | TTTGKTGGGGCGTCCTTAGAC | VIC-ATTGCGATCTCCTGTCCA-MGB |
|  |  | CGCTTGATGTAGCGTCCTTAGAC |  |
| Adenovirus | GCCACGGTGGGGTTTCTAAACTT | GCCCCAGTGGTCTTACATGCACATC | FAM-TGCACCAGACCCGGGCTCAGGTACTCCGA-BQ1 |
| Adenovirus 40/41 | TGCCCGCGCCACCGAT | GAGCCACAGTGGGGTTTCTG | FAM-CCAGGCTGAAGTACG-BQ1 |
| **Bacteria** |  |  |  |
| *Campylobacter jejuni* | ATGCAAACCATAATTGGGTTTCAAC | CGAGTATCAGCAACTTCTTCTACAGCT | NED-TTGCCACCAAAACCAAAACT-MGB |
| *Yersinia enterocolitica* | GCTKGATTGTCAGGAGTTGGTC | ATCCCCCGCAGTTGGCAT | VIC-ACCCGCTAATGAAGCA-MGB |
| *Vibrio cholerae* | CCACTTAGTGGGTCAAACTATATTGTC | ATGCCCCTAATACATCATTAACGTT | FAM-AGCCACTGCACCCAA-MGB |
| *Salmonella spp* | CGGGTTGCGTTATAGGTCTGA | TGAAATACGATGCGAACAACATC | VIC-AATACTGCGCTGCCAGAT-MGB |
| *ETEC-estA* | AAGCATGAATAGTAGCAATTACTGCT | TTAATAGCACCCGGTACAAGCA | NED-AACAACACAATTCAC-MGB |
| *ETEC-eltB* | TCCGGCAGAGGATGGTTACA | CCAGGGTTCTTCTCTCCAAGC | FAM-AGCAGGTTTCCCACCGGATCACC-BQ1 |
| *Shigella spp* | ACCGGCGCTCTGCTCTC | GCAATGTCCTCCAGAATTTCG | JOE-CTGGGCAGGGAAATGTTCCGCC-BQ1 |
|  |  |  |  |
| **Protozoa** |  |  |  |
| *Cryptosporidium parvum/hominis* | CAAATTGATACCGTTTGTCCTTCTG | TGGTGCCATACATTGTTGTCCT | NED-TGTCCTCCTGGATTCA-MGB |
|  |  |  |  |
| **Blood PCR** |  |  |  |
| Malaria | TAATGCCTAGACGTATTCCTGATTATCCAG (1st PCR) | TGTTTGCTTGGGAGCTGTAATCATAATGTG (1st PCR) | |
|  | GAGAATTATGGAGTGGATGGTG (nested PCR) | TGGTAATTGACATCCAATCC (nested PCR) |  |
| Dengue virus | 5´-TCA ATA TGC TGA AAC GCG CGA GAA ACC G | |  |
|  | 5´-CGT CTC AGT GAT CCG GGG G |  |  |
|  | 5´-CGC CAC AAG GGC CAT GAA CAG |  |  |
|  | 5´-TAA CAT CAT CAT GAG ACA GAG C |  |  |
|  | 5´-TGT TGT CTT AAA CAA GAG AGG TC |  |  |
| West Nile fever virus | AGY MGR GCH ATH TGG TWY ATG TGG | TTC CAV CCD GCK GTR TCA TC |  |
| **Blood qPCR** |  |  |  |
| *Rickettsia* spp (all except typhus group) | GTGAATGAAAGATTACACTATTTAT | GTATCTTAGCAATCATTCTAATAGC | 6FAM- CTATTATGCTTGCGGCTGTCGGTTC |
| *Rickettsia felis* | ATGTTCGGGCTTCCGGTATG | CCGATTCAGCAGGTTCTTCAA | 6FAM- GCTGCGGCGGTATTTTAGGAATGGG |
|  | CCCTTTTCGTAACGCTTTGCT | GGGCTAAACCAGGGAAACCT | 6FAM- TGTTCCGGTTTTAACGGCAGATACCCA |
| *Rickettsia* spp ( typhus group) | TGCTTCATGGGCAATGTCTG | TTGAGCATAAAACTGCCCTGCT | 6FAM- CGCTGGATTATCAAAAGAATTAGCACG |
| Chikungunya virus | GGA AAA GTC CTG GAC AGA AAC ATT | ATG TTG GCG TCT CCG TGT CT | FAM - CTT ACA AGC AGT AAT GGC -MGB |
|  | GGA AAA GTC CTG GAC AGA AAC ATC | ATG TTG GCG TCT CCT TGT CTG | FAM - TT ACA AGC GGT GAT GGC - MGB |
| Rift Valley fever virus | AAC TCT CGG ACC CAC TGT TCA A | TTG CGA TCC AGT TTG CTG C | FAM-TGC GGT CCA CTG CT |
| West Nile fever virus | AAT GCC CCG CGT GTT GT | CCG TCR ATC AGG CTC AAC AT | 6FAM-TGG ACT GAA GAG GGC-MGB |
|  |  |  |  |
|  |  |  |  |
