# Supplementary material for: Acute Uncomplicated Febrile Illness in Children Aged 2-59 months in Zanzibar – Aetiologies, Antibiotic Treatment and Outcome
Source: PLoS One. 2016 Jan 28;11(1):e0146054. doi: 10.1371/journal.pone.0146054 (PMC4731140; doi:10.1371/journal.pone.0146054)
Supplement: S2 Table — (DOCX) [file pone.0146054.s005.docx]

| **S2- Table**  **Multiple logistic regression analysis of pathogens detected in nasopharyngeal swabs from patients and controls** | | | | | | |
| --- | --- | --- | --- | --- | --- | --- |
|  | **Unadjusted OR** | **Adjusted OR^a^** | **CI^a^** | **P^a^** |  |  |
| Age (months) | 0.97 | 0.96 | 0.95-0.97 | <0.0001 |  |  |
| Enterovirus | 15.6 | 15.8 | 2.4-130 | 0.005 |  |  |
| Influenza A virus | 5.1 | 8.3 | 2.5-27-4 | 0.001 |  |  |
| Influenza B virus | 6.6 | 10.8 | 3.8-30.4 | <0.0001 |  |  |
| Respiratory Syncytial Virus | 1.8 | 1.9 | 1.2-3.0 | 0.008 |  |  |

^a^ Patient/healthy control was dependent variable, whereas microbial detection (positive/negative), sex and age were independent variables.

Only agents with P < 0.05 were included in the final analysis and presented here. OR, odds ratio; CI, confidence interval.
